# Sterol dysregulation in Smith–Lemli–Opitz syndrome causes astrocyte immune reactivity through microglia crosstalk

**DOI:** 10.1242/dmm.049843

**Published:** 2022-12-16

**Authors:** Bethany A. Freel, Benjamin A. Kelvington, Sonali Sengupta, Malini Mukherjee, Kevin R. Francis

**Affiliations:** ^1^ Basic Biomedical Sciences, University of South Dakota, Vermillion, SD 57069, USA; ^2^ Cellular Therapies and Stem Cell Biology Group, Sanford Research, Sioux Falls, SD 57104, USA; ^3^ Functional Genomics and Bioinformatics Core, Sanford Research, Sioux Falls, SD 57104, USA; ^4^ Department of Pediatrics, Sanford School of Medicine, University of South Dakota, Sioux Falls, SD 57105, USA

**Keywords:** Cholesterol, Astrocyte, *Dhcr7*, Reactivity, Microglia, Smith–Lemli–Opitz syndrome

## Abstract

Owing to the need for *de novo* cholesterol synthesis and cholesterol-enriched structures within the nervous system, cholesterol homeostasis is critical to neurodevelopment. Diseases caused by genetic disruption of cholesterol biosynthesis, such as Smith–Lemli–Opitz syndrome, which is caused by mutations in 7-dehydrocholesterol reductase (*DHCR7*), frequently result in broad neurological deficits. Although astrocytes regulate multiple neural processes ranging from cell migration to network-level communication, immunological activation of astrocytes is a hallmark pathology in many diseases. However, the impact of DHCR7 on astrocyte function and immune activation remains unknown. We demonstrate that astrocytes from *Dhcr7* mutant mice display hallmark signs of reactivity, including increased expression of glial fibrillary acidic protein (GFAP) and cellular hypertrophy. Transcript analyses demonstrate extensive *Dhcr7* astrocyte immune activation, hyper-responsiveness to glutamate stimulation and altered calcium flux. We further determine that the impacts of *Dhcr7* are not astrocyte intrinsic but result from non-cell-autonomous effects of microglia. Our data suggest that astrocyte–microglia crosstalk likely contributes to the neurological phenotypes observed in disorders of cholesterol biosynthesis. Additionally, these data further elucidate a role for cholesterol metabolism within the astrocyte–microglia immune axis, with possible implications in other neurological diseases.

## Teaser

**Summary:** Inhibition of cholesterol biosynthesis in microglia results in reactive microgliosis and subsequently astrogliosis in a mouse model of Smith–Lemli–Opitz syndrome, highlighting the role of lipid metabolism in regulating nervous system health and function.

## INTRODUCTION

Maintenance of cholesterol levels in the brain, the most cholesterol-rich organ in the body, is critical for normal development and function. However, the presence of the blood–brain barrier requires cholesterol in the brain to be synthesized *de novo*, primarily by astrocytes (Jeske and Dietschy, 1980; Mahley, 2016). Although cellular usage of a modified version of the Kandutsch–Russell pathway has also been reported within the brain, cell-specific studies are needed to clarify this finding (Mitsche et al., 2015). Recent work in *Dhcr24* null mice demonstrated that both neurons and astrocytes preferentially use the Bloch pathway (Genaro-Mattos et al., 2019). Disruption of cholesterol homeostasis has also been shown to perturb cell-autonomous and non-cell-autonomous functions, including inhibition of crucial signaling pathways and cellular crosstalk (Bjorkhem and Meaney, 2004; Martin et al., 2014). Additionally, loss of cholesterol homeostasis has been implicated in numerous neurological disorders, including autism spectrum disorder (Tierney et al., 2021). Defining the impact of cholesterol biosynthetic defects on specific cell types within the nervous system is critical to understanding brain development and cholesterol-associated human disease.

As the most numerous cell type in the mammalian brain, astrocytes support neurite outgrowth, neuronal survival, synapse formation and synaptic pruning, all of which are vital during brain development (Chung et al., 2013; Clarke and Barres, 2013). Previous work has also highlighted the downstream impacts of astrocyte immune activation on cellular glutamate uptake and calcium signaling (Agulhon et al., 2012; Haroon et al., 2017; Shigetom, et al., 2019; Vallejo-Illarramendi et al., 2006). Chronic neuroinflammation in neurodevelopmental disorders can disrupt these functions and further impair astrocyte biology. Additionally, astrocytes supply cholesterol to neurons through the transport of apolipoprotein E (APOE) particles (Vance, 2012). Previous work identified a direct impairment of neurite outgrowth and synapse formation when cholesterol biosynthesis was disrupted in astrocytes alone, further highlighting the importance of astrocyte health and function in the context of neurodevelopment (Ferris et al., 2017). As research on neurodevelopmental disorders has progressed, astrocytes have become a key cell type of interest. Previous work identified astrocytic deficits in a variety of neurodevelopmental disorders, including astrocyte immune activation, aberrant calcium signaling, disturbed glutamate uptake, downstream impacts on neurite outgrowth and synaptogenesis, and APOE secretion (Dong et al., 2018; Higashimori et al., 2016; Jacobs and Doering, 2010; Laurence and Fatemi, 2005; Trachtenberg et al., 2012; Vargas et al., 2005). In response to injury or disease, astrocytes commonly mount a neuroinflammatory response, which then induces a reactive cellular state (Sofroniew and Vinters, 2010; Zamanian et al., 2012). Although the purpose of astrocyte activation is to limit tissue damage, growing evidence shows that reactive astrocytes also induce adverse outcomes (Guttenplan et al., 2021; Liddelow and Barres, 2017; Liddelow et al., 2017). Historically, astrocyte activation has been characterized by cellular hypertrophy and an increase in glial fibrillary acidic protein (GFAP) expression; however, recent work has shown that lipid droplet accumulation and the upregulation of key transcripts also occur in reactive astrocytes (Pekny and Nilsson, 2005; Sofroniew, 2009).

Loss of cholesterol homeostasis has been implicated in numerous neurological disorders, including autism spectrum disorder and inborn errors of metabolism such as Smith–Lemli–Opitz syndrome (SLOS) (Tierney et al., 2006). SLOS results from mutations in *DHCR7*, which reduces 7-dehydrocholesterol (7-DHC) to cholesterol in the final step of the cholesterol biosynthesis pathway (Smith et al., 1964). This results in reduced cholesterol levels, accompanied by the accumulation of the precursor 7-DHC. SLOS presents with a wide range of phenotypes including microcephaly, structural malformations, intellectual disability and behavioral issues (Bianconi et al., 2015; Lee et al., 2013). Although the impact of DHCR7 disruption on neuronal dysfunction has been extensively studied, astrocytes and their associated cellular impacts within SLOS have received less attention.

In this study, we demonstrate that dysregulation of cholesterol metabolism induces a reactive state in *Dhcr7* astrocytes and microglia, characterized by both morphological and transcriptional changes. Additionally, co-culture assays determined that reactive microglia drive reactive astrogliosis in *Dhcr7* models. Lastly, reactive astrocytes also display functional deficits in glutamate-dependent calcium signaling. These studies suggest that glial cholesterol biosynthesis is critical for the suppression of reactive gliosis, with relevance to both disorders of cholesterol biosynthesis and neurological disease.

## RESULTS

### Cholesterol-deficient astrocytes display hallmark signs of immune activation

**Fig. 1.**
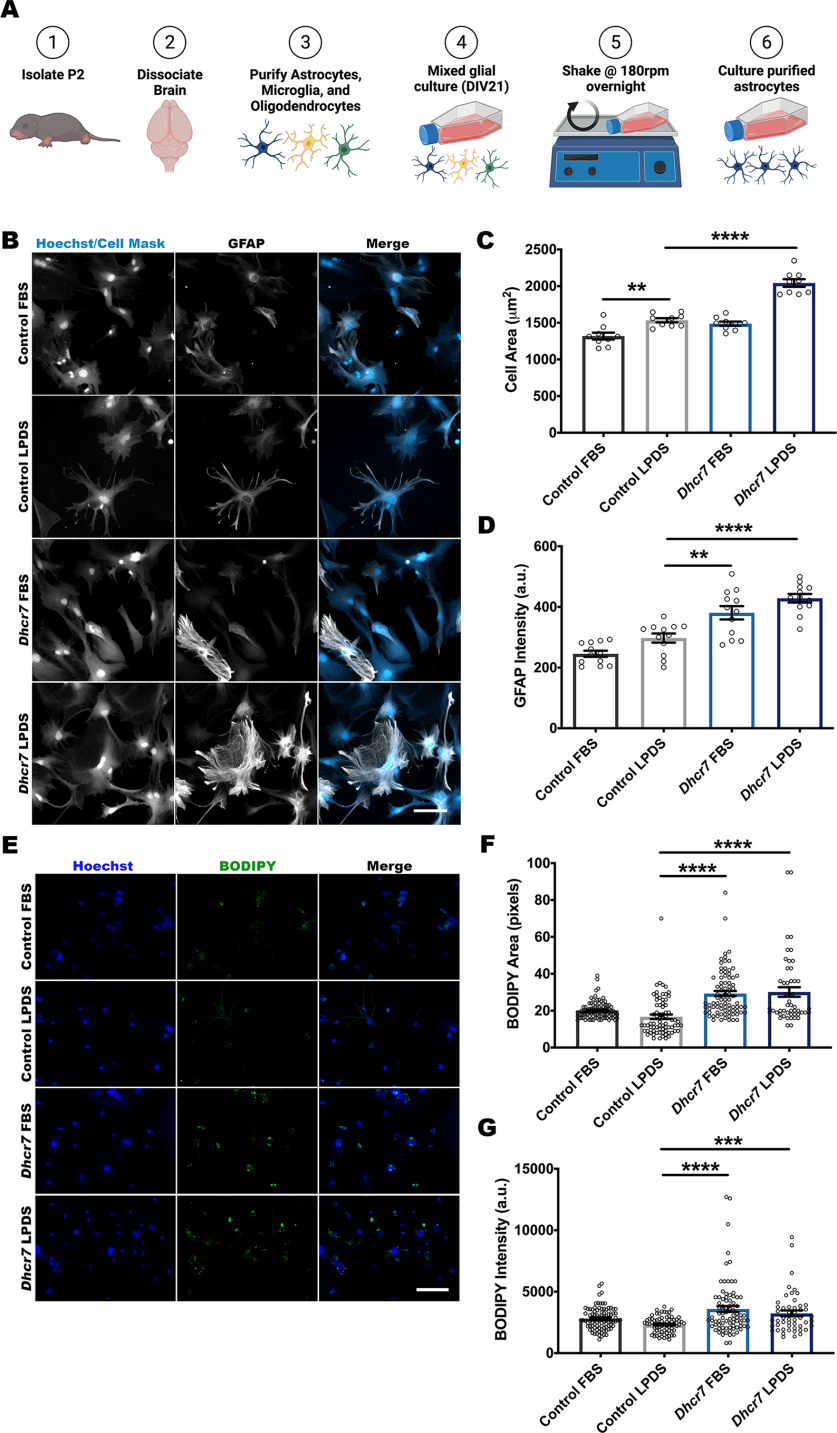
***Dhcr7* mutant astrocytes exhibit hallmark signs of immune activation.** Analysis of purified astrocytes from *Dhcr7* mutant mice shows hallmark signs of astrocyte activation. (A) Schematic of the experimental protocol for primary astrocyte isolation and purification. DIV21, 21 days *in vitro* culture. (B) Representative images showing CellMask (blue) staining and GFAP (white) immunolabeling of control and *Dhcr7* primary astrocytes. Scale bar: 100 µm. (C,D) High-content screening of control and *Dhcr7* primary astrocytes shows cellular hypertrophy and increased GFAP intensity in *Dhcr7* mutant astrocytes following 7 days of culture in LPDS conditions (*n*=9 wells for cell area and *n*=12 wells for GFAP intensity from three biological replicates, mean of 40 images per well). (E) Representative images showing Hoechst 33342 (blue) and BODIPY (green) labeling of control and *Dhcr7* mutant primary astrocytes. Scale bar: 100 µm. (F,G) Analysis of control and *Dhcr7* mutant primary astrocytes shows increased BODIPY intensity and spot area in *Dhcr7* mutant astrocytes (*n*=75 cells per group from three biological replicates, five images per replicate; each data point represents average spot area or intensity per cell). Data show the mean±s.e.m. a.u., arbitrary units. **P<0.01; ***P<0.001; ****P<0.0001 (one-way ANOVA and Dunnett's test versus LPDS control).

Inflammatory competence is a key biological function of astrocytes, and its disruption has been identified in numerous neurological diseases. *Dhcr7*^Δ3-5/T93M^ mutant mice, a hypomorphic mouse model of SLOS, are heterozygous for a targeting vector to disrupt *Dhcr7* via a neomycin insertion, with deletion of coding exons III, IV and part of V, along with a dinucleotide mutation in codon 89 of *Dhcr7*, resulting in an ACA (Thr) to ATG (Met) change (Correa-Cerro et al., 2006; Wassif et al., 2001). This is a silent polymorphism that recapitulates a common point mutation occurring in SLOS patients. Although these mice exhibit reduced disease severity, they do survive past postnatal day (P) 1, which is critical for the efficient isolation of primary glia. Following isolation of mixed glia from control (*Dhcr7*^WT/T93M^) and *Dhcr7*^Δ3-5/T93M^ littermates (hereafter referred to as *Dhcr7* mice) and *in vitro* culture, astrocytes were purified by mechanical isolation (Fig. 1A) and culture purity validated with immunolabeling of cellular markers (Fig. S1A). To determine the impact of *Dhcr7* disruption, astrocytes were cultured in either cholesterol-replete conditions with fetal bovine serum (FBS) or under biochemical stress in lipoprotein-deficient (cholesterol-depleted) serum (LPDS) conditions. Culturing cells in LPDS conditions requires them to synthesize their own cholesterol, rather than taking it up from the medium, as is the case when cultured in FBS conditions. When *Dhcr7*-deficient cells are cultured in LPDS conditions, reduced cholesterol levels and the accumulation of the precursor 7-DHC can be detected. Following 7 days of LPDS culture, *Dhcr7* astrocytes exhibited the expected biochemical phenotype with a reduction in cholesterol and the detection of 7-DHC when analyzed by gas chromatography/mass spectrometry (GC/MS) (Fig. S1B,C). This phenotype is caused by reduced activity of the Dhcr7 enzyme to adequately convert 7-DHC to cholesterol when cultured in cholesterol-deficient conditions. *Dhcr7* astrocytes were immunolabeled with GFAP and CellMask Blue stain (Fig. 1B), displaying a significant increase in both cellular area and GFAP intensity (Fig. 1C,D), suggestive of astrocyte reactivity. *Dhcr7* astrocytes also displayed another reactive cellular phenotype, accumulation of BODIPY-labeled lipid droplets (Fig. 1E). Both BODIPY spot intensity (Fig. 1G) and spot size (Fig. 1F) were increased in *Dhcr7* mutant samples. To validate these findings *in vivo*, brains from P7 and P30 mice were labeled with GFAP to quantify changes in intensity (Fig. 3A,E). Analysis of GFAP intensity showed an increase at both timepoints (Fig. 3B,F). These data demonstrate that *Dhcr7* loss results in a morphological change accompanied by lipid droplet accumulation that is characteristic of astrocyte immune reactivity.

### *Dhcr7* mutant astrocytes exhibit a robust transcriptional immune activation

**Fig. 2.**
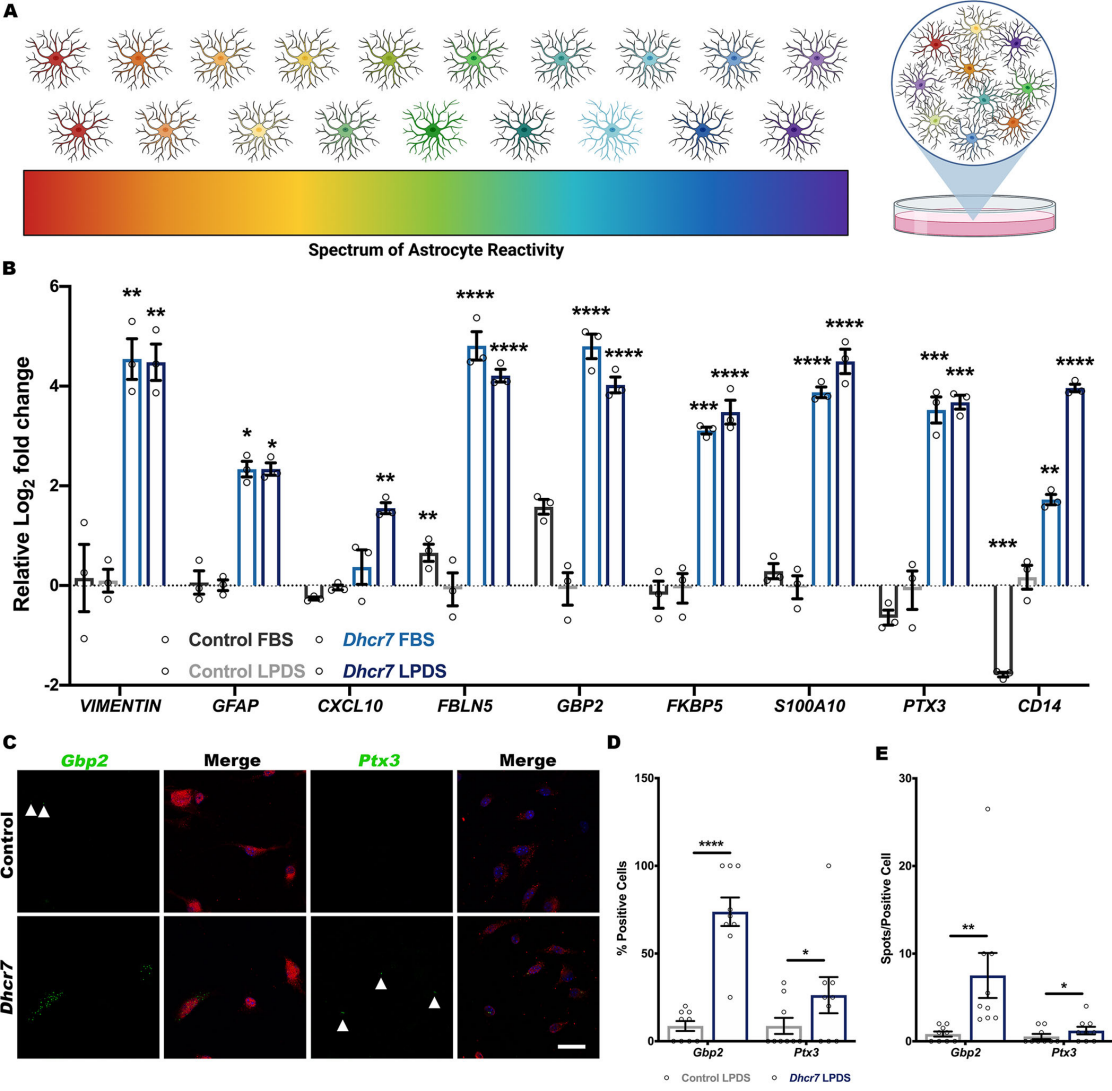
***Dhcr7* astrocytes exhibit a transcriptional signature encompassing reactive astrocyte subtypes.** *Dhcr7* astrocytes display increased transcripts for neurotoxic and neuroprotective reactive subtypes. (A) Spectrum of reactive astrocyte states, which can exist as a mixed population. (B) The expression of reactive transcripts is increased in *Dhcr7* astrocytes independent of environmental cholesterol (one-way ANOVA and Dunnett's test versus LPDS control; *n*=3 biological replicates; each data point represents the average of three technical replicates). (C) *In situ* hybridization experiments with probes specific to reactive astrocyte transcripts (*Gbp2*, green; *Ptx3*, pseudo-colored green for visibility) and GFAP (red) immunolabeling of control and *Dhcr7* astrocytes cultured in LPDS conditions are suggestive of different reactive astrocyte states. Hoechst 33342 counterstain is in blue. Scale bar: 25 µm. (D,E) *In situ* hybridization demonstrated both increased reactive subtype-positive cells and increased expression of transcripts per cell in *Dhcr7* astrocytes cultured in LPDS conditions (two-tailed unpaired *t*-test; *n*=9 images from three biological replicates). Data show the mean±s.e.m. *P<0.05; **P<0.01; ***P<0.001; ****P<0.0001.

Previous work has determined that reactive astrocytes can adopt unique transcriptomes in different disease models, which can disrupt astrocyte homeostasis or induce protective functions (Escartin et al., 2021; Itoh et al., 2018; Zamanian et al., 2012). Recent work has shown that reactive astrocyte states are complex and that they can exist as mixed populations or even with subsets of cells expressing transcripts, suggestive of multiple cellular subtypes (Al-Dalahmah et al., 2020; Ceyzeriat et al., 2018; Das et al., 2020; Diaz-Castro et al., 2019; Grubman et al., 2019; Liddelow and Barres, 2017; Zhou et al., 2020) (Fig. 2A). *Dhcr7* mutant astrocytes exhibited robust increases in expression of previously identified reactive astrocyte transcripts (Fig. 2B; Table S2) (Liddelow et al., 2017). To determine whether these transcriptional changes signified distinct reactive subtypes that co-exist or co-expression within individual cells, *in situ* hybridization assay was performed to detect target RNA within control and *Dhcr7* astrocytes cultured in LPDS conditions (Fig. 2C). Two of the markers analyzed by quantitative real-time PCR (qRT-PCR) assays, guanylate-binding protein 2 (*Gbp2*) and pentraxin 3 (*Ptx3*), were chosen for downstream validation. Gbp2 is implicated in interferon γ signaling and the innate immune system where it functions as a GTPase. Ptx3 is induced by inflammatory cytokines in response to inflammatory stimuli and is involved in regulating inflammation. Increased expression of both *Gbp*2 and *Ptx3* reactive transcripts was identified in *Dhcr*7 astrocytes, although distinct reactive subtypes appeared to exist in *Dhcr7* astrocyte cultures (Fig. 2D,E).

**Fig. 3.**
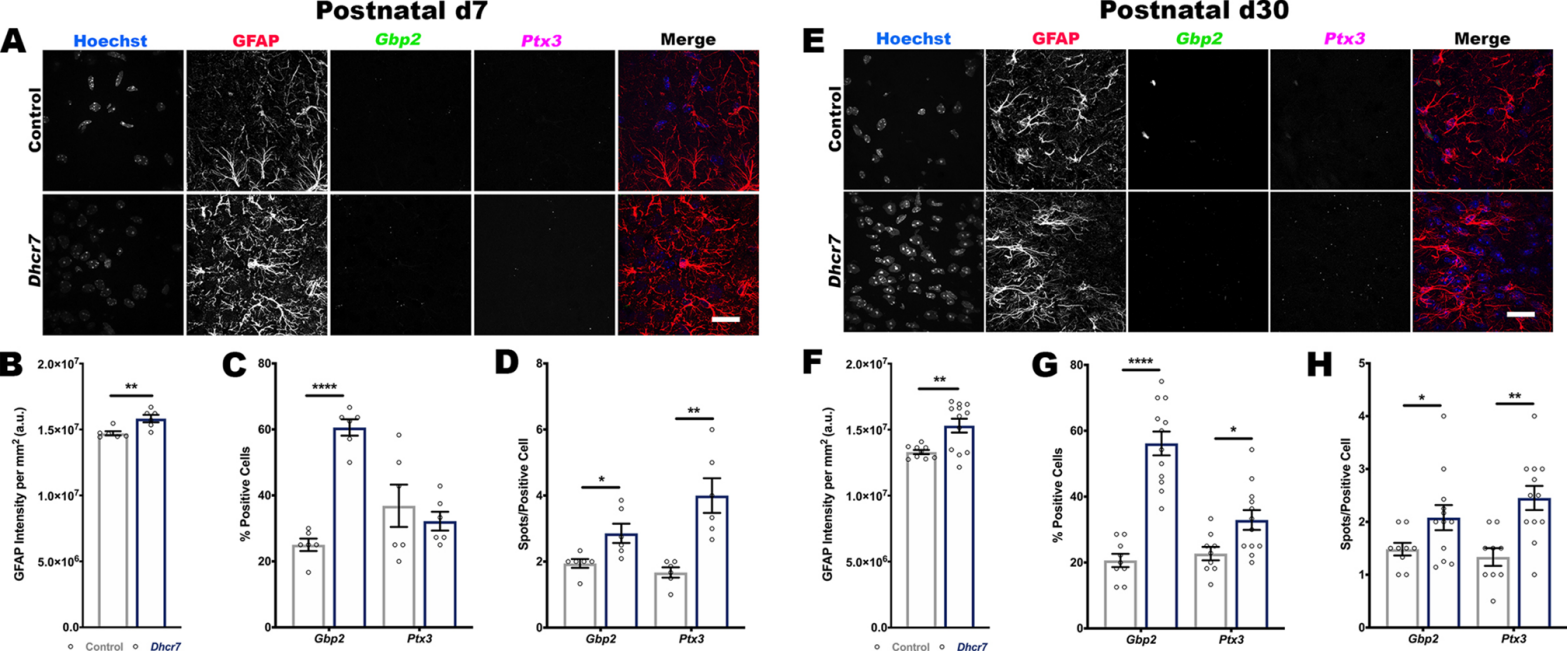
***In vivo* RNAscope demonstrates increased expression of transcripts indicative of reactive astrocyte subtypes.** Transcripts indicative of reactive astrocyte subtypes were increased in the cortex of P7 and P30 *Dhcr7* mice. (A,E) Representative Hoechst 33342 nuclear staining (blue) with GFAP (red), *Gbp2* (green) and *Ptx3* (pink) immunolabeling of control and *Dhcr7* P7 (A) or P30 (E) mouse brain sections. Scale bars: 25 µm. (B,F) Analysis of GFAP intensity normalized to the area of the image field shows increased intensity in the *Dhcr7* cortex at P7 (B) and P30 (F). (C,D,G,H) Both the number of *Gbp2*^+^ and *Ptx3*^+^ cells and the expression of *Gbp2* and *Ptx3* were increased in the P7 (C,D) and P30 (G,H) *Dhcr7* cortex (two-tailed unpaired *t*-test versus control; *n*=9 images from three biological replicates). Data show the mean±s.e.m. a.u., arbitrary units. *P<0.05; **P<0.01; ****P<0.0001.

To determine whether reactive astrocyte subtypes were also present *in vivo*, RNA expression was also analyzed in the cerebral cortex of P7 and P30 mice (Fig. 3A,E). Increased expression of *Gbp2* and *Ptx3* was detected at both timepoints in *Dhcr7* mice *in vivo* (Fig. 3C,D,G,H). These data demonstrate that *Dhcr7* astrocytes exhibit a characteristic transcriptional signature that is suggestive of a mixed population of reactive subtypes.

### *Dhcr7* mutant astrocytes are hyper-responsive to glutamate but not to ATP

**Fig. 4.**
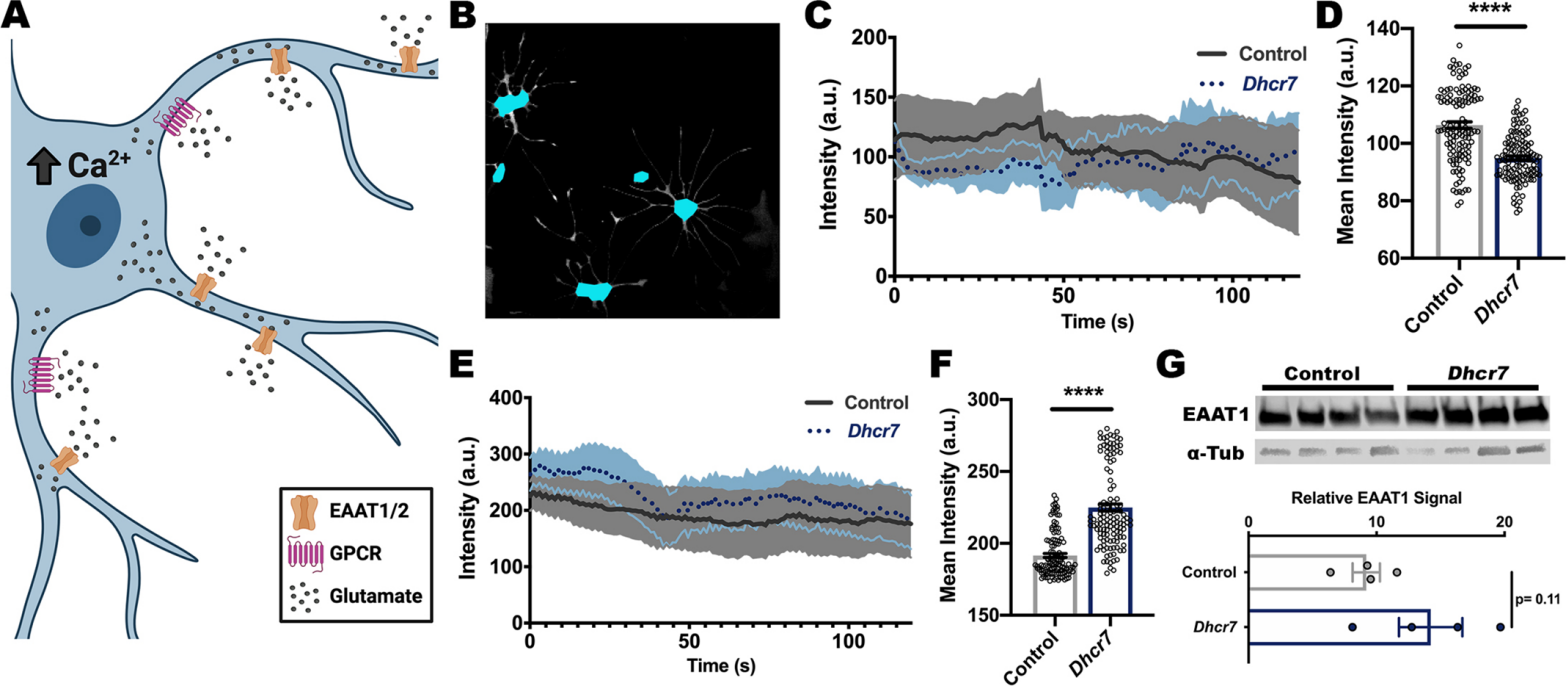
***Dhcr7* astrocytes exhibit hyper-reactive glutamate responsivity.** Following culture in LPDS conditions, *Dhcr7* astrocytes exhibit a hyperactive response to glutamate stimulation. (A) Schematic showing astrocyte response to glutamate uptake through increased calcium signaling. (B) Representative image of cellular segmentation for calcium analysis. (C) Quantitation of calcium imaging shows reduced baseline activity in *Dhcr7* astrocytes. (D) Mean calcium intensity highlights decreased calcium activity in *Dhcr7* astrocytes prior to stimulation (*n*=720 from six recordings from each group; each data point represents the average intensity of replicates over time). (E) Quantitation of calcium imaging shows hyperactive response to glutamate stimulation in *Dhcr7* astrocytes. (F) Mean calcium intensity shows increased calcium activity in *Dhcr7* astrocytes following glutamate stimulation (*n*=360 from three recordings per group; each data point represents the average intensity of replicates over time). (G) Western blot analysis of EAAT1 (GLAST1) in brain lysates of control and *Dhcr7* mice (*n*=4 biological replicates). Data show the mean±s.e.m. a.u., arbitrary units. ****P<0.0001 (two-tailed unpaired *t*-test).

Changes in neurotransmitter release from the synapse is known to induce calcium signaling in astrocytes (Fig. 4A). Given the role of calcium response in cellular signaling, this disruption can contribute to further astrocytic dysfunction (Bazargani and Attwell, 2016). Additionally, this calcium response can trigger many different cellular pathways, including gliotransmitter release. Gliotransmitters have been shown to act on neurons and vascular smooth muscles to regulate synapse release and blood flow (Bazargani and Attwell, 2016; Parpura et al., 2011). To determine whether reactive *Dhcr7* mutant astrocytes displayed changes in calcium signaling, live-cell imaging was performed and intensity changes within the soma were recorded (Fig. 4B). Astrocytes were incubated with Fluo-4 AM to label free intracellular calcium and their activity was recorded for 2 min. Baseline activity was recorded prior to the addition of any stimulant. *Dhcr7* mutant astrocytes displayed reduced baseline activity in comparison to controls (Fig. 4C,D). Additional analysis of *Trpc4* expression, a key regulator of store-operated calcium entry, showed a significant reduction in *Dhcr7* mutant mice (Fig. S2A). This is consistent with a role for *Trpc4* in regulating astrocyte calcium signaling in other autism spectrum disorders (Dong et al., 2018). To stimulate astrocytic calcium signaling, cells were stimulated with 3 µM of glutamate or 3 µM of ATP, and the activity was recorded for an additional 2 min immediately following the addition of the stimulant (Lundin et al., 2018). *Dhcr7* mutant astrocytes exhibited a hyperactive calcium response following glutamate stimulation (Fig. 4E,F). This hyper-responsive phenotype was not recapitulated following stimulation with ATP, suggesting the presence of a glutamate-specific mechanism (Fig. S2B,C). The clearance of excess glutamate by excitatory amino acid transporters (EAATs) is a key cellular function of astrocytes, with EAAT1 (GLAST1 or SLC1A3) and EAAT2 (GLT-1 or SLC1A2) being the predominant isoforms present in astrocytes (Gegelashvili et al., 2000; Mahmoud et al., 2019; Parpura et al., 1994). Previous work has shown that cholesterol homeostasis is required for glutamate uptake, with reduced cholesterol resulting in reduced uptake (Butchbach et al., 2004). *Dhcr7* mutant mice also displayed an increase in EAAT1 protein expression in brain lysates (Fig. 4G). These data suggest that *Dhcr7* astrocytes exhibit impaired function, likely due to immune activation.

### Pharmacological inhibition of cholesterol synthesis in astrocytes does not recapitulate their reactive state

**Fig. 5.**
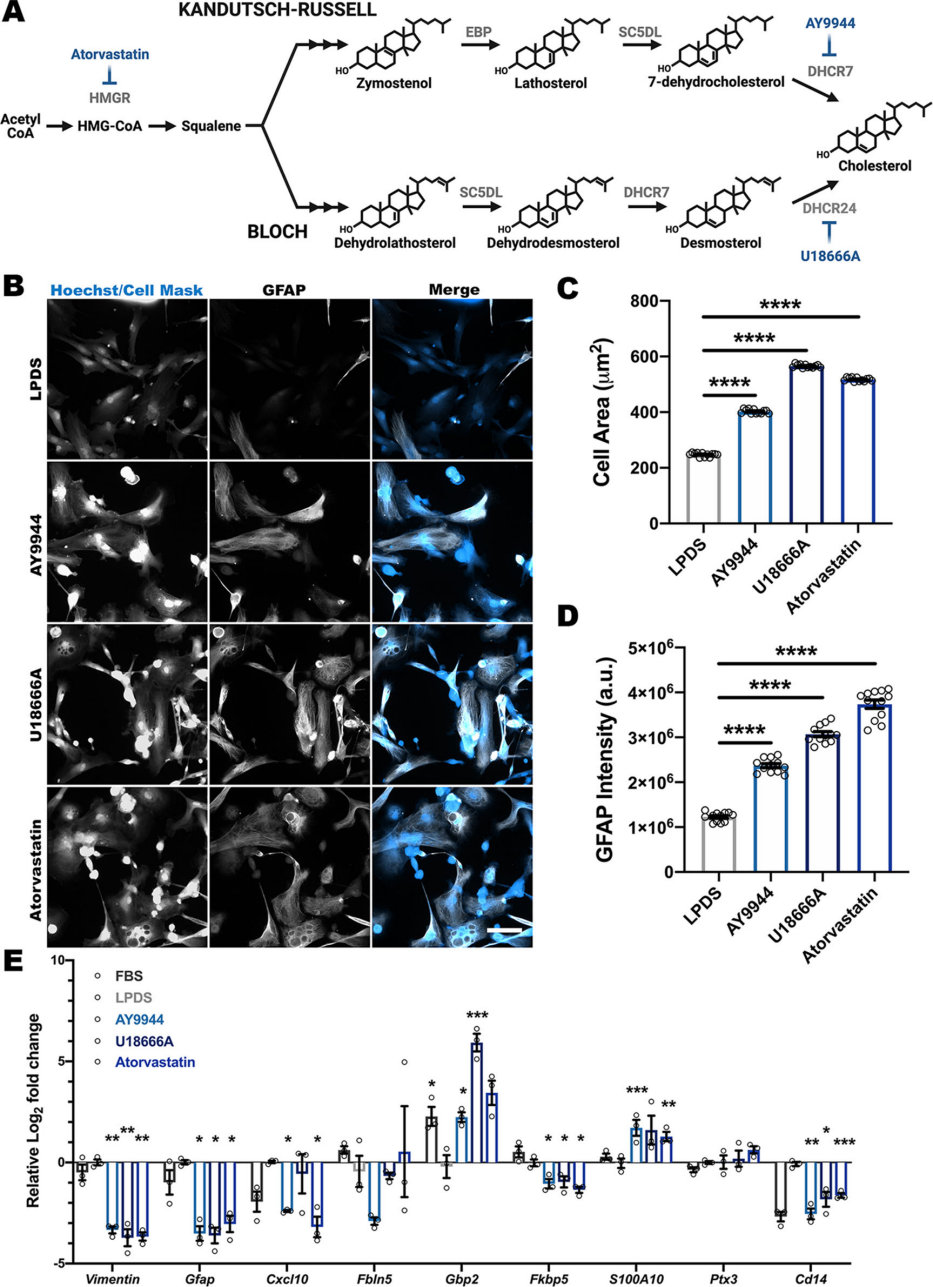
**Pharmacological inhibition of cholesterol synthesis does not recapitulate reactive states observed in *Dhcr7* astrocytes.** Although pharmacological models of SLOS exhibit a morphological change similar to those of genetic models, transcriptional measures of reactivity do not align between the models. (A) Schematic of the cholesterol biosynthesis pathway and pharmacological inhibitors (blue) used to target specific enzymes (gray). (B) CellMask (blue) staining and GFAP (white) immunolabeling of control primary astrocytes. Scale bar: 100 µm. (C,D) Treatment of control astrocytes with cholesterol biosynthesis inhibitors induced cellular hypertrophy and increased GFAP intensity (*n*=12 wells from three biological replicates, mean of 40 images per well). (E) qRT-PCR analysis demonstrated pharmacological inhibition of cholesterol biosynthesis results in muted astrocyte reactivity (*n*=3 biological replicates; each data point represents the average of three technical replicates). Data show the mean±s.e.m. a.u., arbitrary units. *P<0.05, **P<0.01; ***P<0.001; ****P<0.0001 (one-way ANOVA and Dunnett's test versus vehicle-treated LPDS control).

To determine whether the immune activation observed in *Dhcr7* astrocytes was dependent upon intrinsic astrocyte effects, control astrocytes were treated with small-molecule inhibitors of cholesterol biosynthesis and analyzed for reactive morphology and transcript changes (Fig. 5A; Fig. S3A,B). A dose response was performed to determine optimal dosing for primary astrocytes and results were validated with GC/MS (Fig. S3A). Although pharmacological inhibition of cholesterol synthesis resulted in hallmark morphological changes observed upon astrocyte activation (Fig. 5B), including cellular hypertrophy and increased GFAP intensity (Fig. 5C,D), the expression of reactive transcripts was not consistent with our findings in *Dhcr7* astrocytes (Fig. 5E). Additionally, atorvastatin-treated astrocytes trended similarly to AY9944-treated astrocytes for both the immunocytochemistry and qRT-PCR assays (Fig. 5B-E), suggesting that reduction of cholesterol plays a larger role in these cellular changes than does accumulation of 7-DHC. To confirm that this was not a species-specific difference, inhibition of cholesterol synthesis in control human primary astrocytes showed comparable biochemical defects (Fig. S4A) and morphological changes similar to those observed in control mouse astrocytes treated with small-molecule inhibitors of cholesterol synthesis (Fig. S4B-D). These data suggest that *Dhcr7* astrocyte reactivity is likely not astrocyte intrinsic but the result of exposure to other factors or cell types.

### *Dhcr7* microglia also exhibit hallmark signs of immune reactivity

**Fig. 6.**
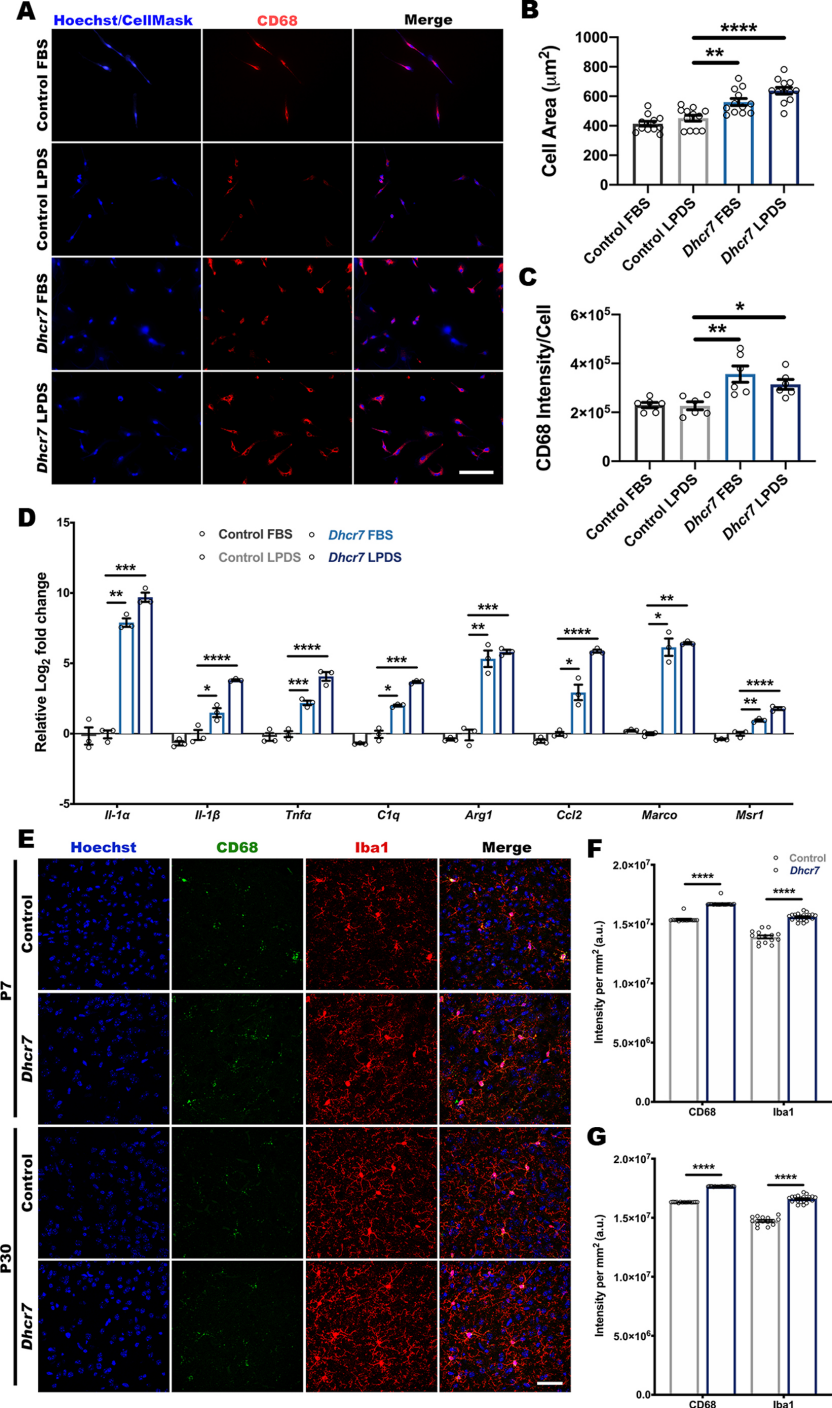
***Dhcr7* microglia exhibit immune activation and markers of reactivity.** Purified microglia from *Dhcr7* mutant mice display hallmark signs of activation. (A) CellMask (blue) staining and CD68 (red) immunolabeling of control and *Dhcr7* microglia. Scale bar: 100 µm. (B,C) High-content analyses showed cellular hypertrophy and increased CD68 expression/cell in *Dhcr7* microglia following culture in LPDS conditions (one-way ANOVA and Dunnett's test versus LPDS control; *n*=12 images for cell area and *n*=6 images for CD68 intensity from three biological replicates). (D) qRT-PCR analysis shows increased expression of reactive transcripts in *Dhcr7* mutant microglia samples (one-way ANOVA and Dunnett's test versus LPDS control; *n*=3 biological replicates; each data point represents the average of three technical replicates). (E) CD68 and Iba1 immunolabeling of P7 and P30 brain sections (cortex) of control and *Dhcr7* mice. Scale bar: 25 µm. (F,G) Analysis of CD68 and Iba1 intensity normalized to the area of the image field shows increased intensity in *Dhcr7* mutant brains at both timepoints (two-tailed unpaired *t*-test; *n*=15 images from three biological replicates). Data show the mean±s.e.m. a.u., arbitrary units. *P<0.05; **P<0.01; ***P<0.001; ****P<0.0001.

Given that primary glia were isolated as a mixed population and co-cultured prior to astrocyte purification, *Dhcr7* astrocytes were therefore exposed to *Dhcr7* microglia *in vitro*. As the resident immune cell within the brain, microglia become activated by injury or disease and can contribute to a reactive astrocyte state (Jha et al., 2019; Liddelow et al., 2017; Matejuk and Ransohoff, 2020; Yang et al., 2020). Microglia display cellular hypertrophy and increased CD68 expression when activated (Jurga et al., 2020). As pharmacological cholesterol inhibition did not recapitulate the reactivity observed in *Dhcr7* astrocytes, we next sought to determine the activation state of *Dhcr7* microglia. Following purification of microglia from mixed glial cultures, immunolabeling for CD68 and CellMask Blue staining showed both increased cell area and CD68 expression per cell in *Dhcr7* microglia, regardless of culture conditions (Fig. 6A-C). Previous work has determined that reactive microglia secrete Il-1α, Tnfα and C1q, which are sufficient and required to induce reactive astrocytes (Liddelow et al., 2017). *Dhcr7* microglia exhibited a transcriptional signature indicative of reactivity (Fig. 6D; Table S2) coupled with increased BODIPY accumulation (Fig. S5A-C) (Liddelow et al., 2017). To validate these findings *in vivo*, brains from P7 and P30 mice were labeled with CD68 and Iba1 (or Aif1) to quantify changes in intensity (Fig. 6E). Analysis of CD68 and Iba1 intensity showed an increase at both timepoints (Fig. 6F,G). Based upon these findings, reactive *Dhcr7* microglia might be an impactful contributor to astrocyte reactivity within this model.

### Mixed-species co-culture between astrocyte and microglia demonstrates that *Dhcr7* microglia drive astrocyte activation

**Fig. 7.**
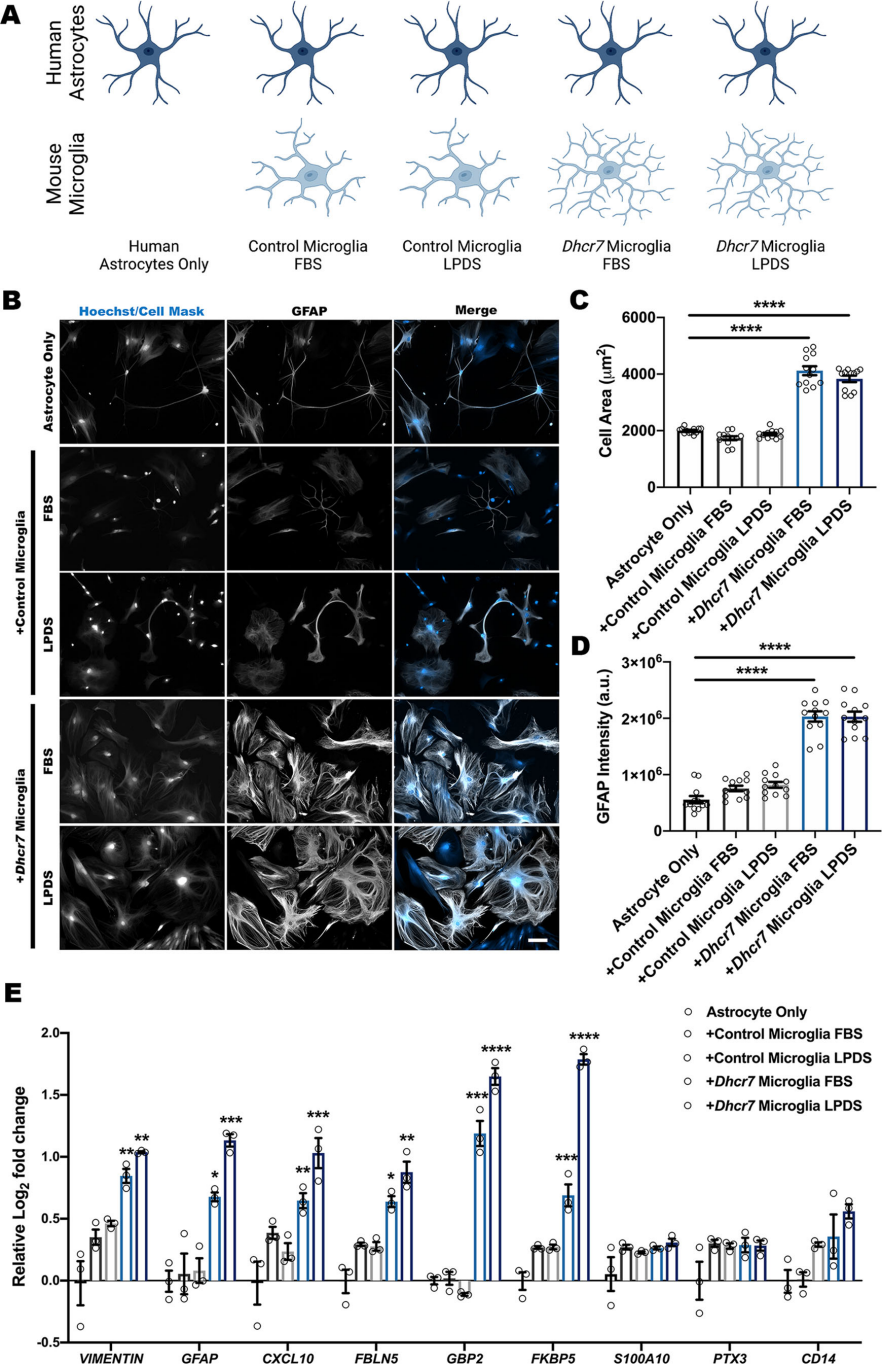
***Dhcr7* mutant microglia drive astrocyte immune activation.** Astrocyte-microglia co-culture assays highlight the impact of reactive microglia on astrocyte activation. (A) Schematic showing the experimental combinations of human astrocytes and mouse microglia. (B) Representative images showing CellMask (blue) staining and GFAP (white) immunolabeling of control human primary astrocytes. Scale bar: 100 µm. (C,D) High-content analyses of co-cultured mouse microglia and human primary astrocytes showed cellular hypertrophy and increased GFAP intensity in astrocytes following *Dhcr7* mutant microglia exposure (*n*=12 images from three biological replicates). (E) qRT-PCR analysis showed increased expression of some, but not all, reactive transcripts in astrocytes co-cultured with *Dhcr7* mutant microglia (*n*=3 biological replicates; each data point represents the average of three technical replicates). Data show the mean±s.e.m. a.u., arbitrary units. *P<0.05; **P<0.01; ***P<0.001; ****P<0.0001 (one-way ANOVA and Dunnett's test relative to astrocyte-only control).

Astrocyte–microglia crosstalk is crucial in maintaining brain homeostasis and functions as a regulatory mechanism in neurological disease, and activated microglia have been shown to induce reactive astrocytes (Jha et al., 2019; Liddelow et al., 2017; Matejuk and Ransohoff, 2020). To determine the impact of *Dhcr7* mutant microglia in driving a reactive astrocyte state, control or *Dhcr7* microglia were co-cultured with control human astrocytes (Fig. 7A). Following co-culture and immunolabeling with GFAP, quantitative analyses showed increased cell area and GFAP intensity in human astrocytes co-cultured with *Dhcr7* microglia (Fig. 7B-D). Consistent with this reactive state, human primary astrocytes cultured with *Dhcr7* mutant microglia also displayed increased accumulation of BODIPY-labeled lipid droplets (Fig. S6A-C). Human astrocytes were used so that human-specific primers corresponding to reactive astrocyte transcripts (Table S3) could be designed (Singh et al., 2022). Using this method, human astrocytes exhibited increased expression of reactive astrocyte transcripts following co-culture with *Dhcr7* microglia (Fig. 7E). These data demonstrate a direct role for microglia in driving glial reactivity following *Dhcr7* disruption, suggesting that microglia cholesterol biosynthesis is required to suppress immune reactivity.

## DISCUSSION

**Fig. 8.**
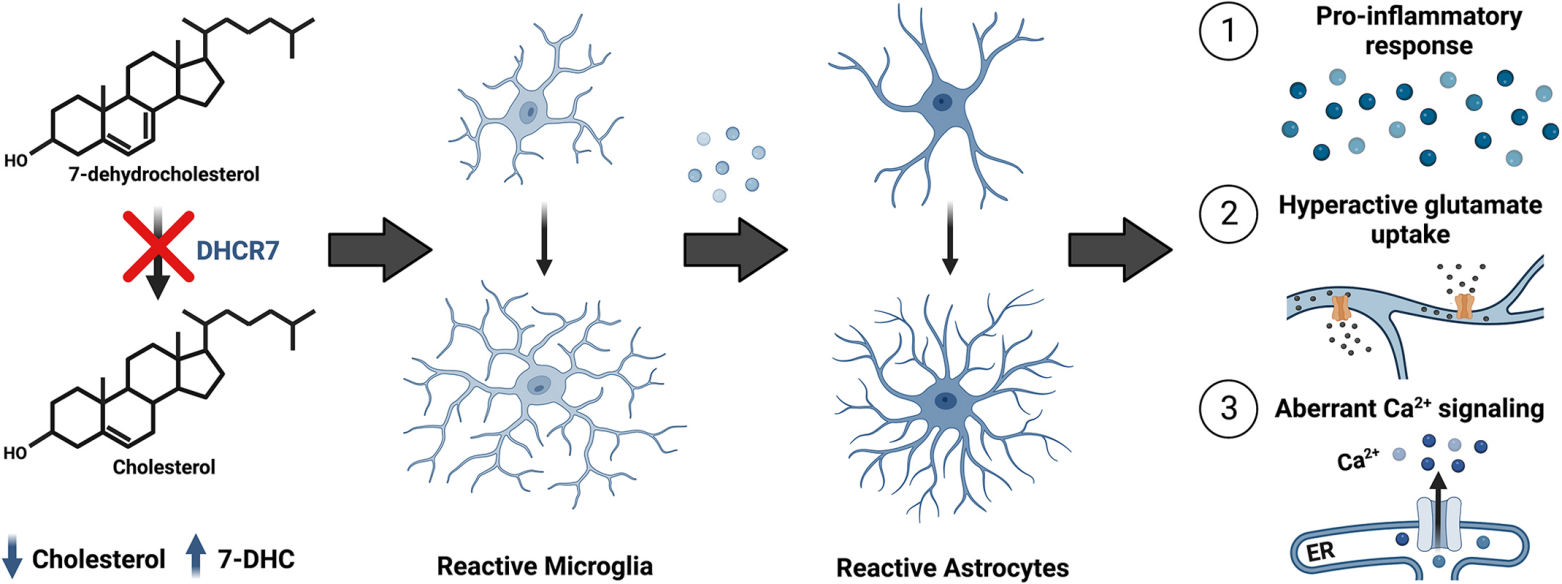
**Cholesterol synthesis disruption in microglia drives reactive gliosis in Smith–Lemli–Opitz syndrome.** Inhibition of *Dhcr7* inhibits microglia cholesterol synthesis and induces reactive astrogliosis, resulting in aberrant astrocyte function. Future work will be needed to detail the intracellular microglial response to cholesterol inhibition, as well as possible external factors contributing to the overall reactive phenotypes observed. ER, endoplasmic reticulum.

Herein, we have presented evidence supporting a role for cholesterol homeostasis in mitigating microglial-induced astrocyte immune activation. Our findings suggest that disruption of cholesterol synthesis triggers a stress response in microglia, causing microglia reactivity. Reactive *Dhcr7* microglia then drive astrocyte activation, disturbing downstream astrocyte functionality, including glutamate responsiveness and calcium activity. Use of co-culture models demonstrated that astrocyte activation primarily occurs due to *Dhcr7* microglia, providing novel evidence that glial activity is regulated by *Dhcr7*. These data suggest a previously unreported regulatory mechanism by which cholesterol homeostasis impacts astroglia–immune connectivity and function, with implications for neurological diseases associated with cholesterol disruption, such as SLOS (Fig. 8). Our work, which suggests a critical role for microglia cholesterol synthesis in promoting normal microglia function, agrees with recently published work in multiple sclerosis and Alzheimer's disease (Berghoff et al., 2021; Tcw et al., 2022). Although the exact mechanisms of microglial cholesterol synthesis are unknown, the activation of the nuclear activity of liver X receptor directly by sterols or sterol metabolites might be critical for this process in SLOS and other neurological diseases (Berghoff et al., 2021; Endo-Umeda et al., 2014). Additional studies will be needed to fully define the pathways and molecular interactors involved.

The interconnectivity between astrocytes and microglia is a burgeoning field with relevance to various neurological diseases. Astrocytes respond to injury and disease by mounting a neuroinflammatory response, inducing a reactive cellular state with transcriptional signatures unique to reactive astrocyte subtypes (Clarke et al., 2018; Darmanis et al., 2015; Liddelow and Barres, 2017; Pekny and Nilsson, 2005; Sofroniew, 2009; Sofroniew and Vinters, 2010; Zhang et al., 2016). A critical function of astrocytes is their response to neurotransmitter release at synapses via intracellular and extracellular astrocytic calcium signaling. Impaired astrocyte function, including altered calcium signaling and glutamate responsivity that we demonstrated, are also indicators of reactivity and dysfunctional astrocyte biology (Agulhon et al., 2012; Haroon et al., 2017). Astrocytic calcium signaling and flux from internal calcium stores is associated with glutamate uptake and cellular signaling mechanisms. Regulation of glutamate metabolism by astrocytes is an important modulator of synaptic activity with additional impacts on blood flow through vascular smooth muscle cells (Bazargani and Attwell, 2016; Parpura et al., 2011). As the resident immune cells of the brain, microglia function in the surveillance of the brain milieu to respond to injury or disease (Andjelkovic et al., 1998; Li and Barres, 2018). Microglia likely drive the bidirectional conversation with astrocytes, either inducing a neurotoxic or neuroprotective astrocyte response while also regulating astrocyte innate immune function (Jha et al., 2019; Matejuk and Ransohoff, 2020). In return, astrocytes regulate microglia function, such as phagocytosis and motility, through secreted molecules (Jha et al., 2019; Matejuk and Ransohoff, 2020). Astrocyte–microglia communication contributes to neurological phenotypes in multiple diseases (Jha et al., 2019; Lian et al., 2016; Matejuk and Ransohoff, 2020; Yang et al., 2020). However, our current understanding of what factors contribute to pathological induction of this cellular interaction remain largely unknown. Further study of this immune–glia axis will elucidate the role that this cellular interaction plays within neurological disease.

The impact of lipid metabolism on brain development and function within neurological disease is also an emerging area for discovery. Based upon the transcriptional profiles identified in *Dhcr7* mutant astrocytes and microglia, cholesterol biosynthesis and metabolism appear critical for the suppression of reactive glial states. Additionally, the identification of mixed populations of astrocyte subtypes (based upon the expression patterns of transcripts consistent with different reactive astrocyte states) within this developmental disorder is a unique finding. Although microglia appear to induce a reactive astrocyte state, only some reactive transcripts are changed, suggesting that a different subset of reactive astrocytes is present and is induced by a different stimulus.

Our work also extends previous studies detailing the impact of cholesterol biosynthesis on microglia reactivity. Previous work demonstrated that inhibition of HMG-CoA reductase by statin therapy inhibited microglia-induced neuroinflammation through attenuated lipopolysaccharide-induced cytokine release, suppressed reactive oxygen species generation, and increased M2/M1 macrophage ratio (McFarland et al., 2017; Xu et al., 2017), possibly through suppression of toll-like receptor (TLR) and/or Notch signaling pathways (Han et al., 2018; Wu et al., 2018). M1 reactivity has been shown to rely on several signaling pathways, including TLR and Notch signaling. Toll-like receptors TLR2 and TLR4 are highly expressed in microglia and regulate M1 reactivity (Cui et al., 2020). Notch signaling is implicated in M1 polarization through transcriptional activation of genes pertaining to cellular proliferation, death and polarization by the Notch intracellular domain (Guo et al., 2022; Wu et al., 2018). Additional studies are needed to detail how various disruption paradigms of cholesterol metabolism impact pathways that require microglia, such as TLR or Notch signaling, as well as how signaling changes coincide with microglia activity.

A critical unanswered question from our work is how *Dhcr7* disruption causes microglia activation. Are microglia self-reactive due to endogenous cholesterol dysfunction or is increased reactivity in response to external stimuli? Although microglia typically become reactive in response to neuronal damage or loss, limited work has analyzed endogenous microglia lipid metabolism to ascertain its importance. A detailed comparison of *Dhcr7* microglia to statin- and/or AY9944-treated microglia at the morphological, transcriptional and functional levels will be required. For instance, direct analyses of microglia from targeted brain regions via immunopanning or single-cell RNA-sequencing will detail microglia heterogeneity and reactive identity.

The *Dhcr7* animal models used here have historically been used to detail cholesterol-associated neuronal deficits in SLOS. Various structural and functional deficits resulting from *Dhcr7* disruption have been previously described in neurons and neural progenitors using these animals and cellular models (Francis et al., 2016; Jiang et al., 2010; Korade et al., 2010; Xu et al., 2013). Previous work identified a direct impairment of neurite outgrowth and synapse formation when cholesterol biosynthesis was disrupted in astrocytes alone, further highlighting the importance of astrocyte health and function in the context of neurodevelopmental disorders (Mulder, 2009; Pfrieger and Ungerer, 2011). However, the impacts of cholesterol biosynthesis on astroglia biology have only recently been explored (Genaro-Mattos et al., 2019). Astrocytes support neurite outgrowth, synapse formation, synaptic pruning and neuronal survival, all of which are vital during brain development (Chung et al., 2013; Clarke and Barres, 2013; Liddelow and Barres, 2015; Sofroniew and Vinters, 2010). A recently developed mouse carrying LoxP-flanked *Dhcr7* should enable the discernment of cell-type-specific impacts of *Dhcr7* deletion (Kanuri et al., 2020). Subsequent studies analyzing astrocyte- or microglia-specific deletion of *Dhcr7* will allow us to define the contribution of each cell type to the developmental and functional deficits within the *Dhcr7-*deficient central nervous system and also improve our understanding of SLOS pathogenesis.

In summary, we have demonstrated that *Dhcr7* mutant astrocytes are reactive in response to their exposure to reactive microglia. This reactive astrocyte state is coupled with downstream impacts on astrocytic function, including glutamate uptake and calcium signaling. Our data suggest that *Dhcr7* disruption alters astrocyte–microglia crosstalk, likely driving pathological phenotypes that can affect neuronal function and overall brain health. Additional studies detailing the impact of cholesterol metabolism on glial biology, immune activation and the complexities of cellular crosstalk in the brain are needed.

## MATERIALS AND METHODS

### Animals and housing

*Dhcr7*^Δ3-5/T93M^ mice (generated by mating *Dhcr7*^WT/Δ3-5^ and *Dhcr7*^T93M/T93M^ mice, kindly provided by Dr Forbes Porter, Eunice Kennedy Shriver National Institute of Child Health and Human Development) are heterozygous for a *Dhcr7* targeting vector containing a neomycin insertion, which results in deletion of *Dhcr7* exons III, IV and part of V, along with a dinucleotide mutation in codon 89 (Correa-Cerro et al., 2006; Wassif et al., 2001). As previously reported, *Dhcr7*^WT/T93M^ mice are used as controls for experiments with *Dhcr7*^Δ3-5/T93M^ mice (Correa-Cerro et al., 2006). Mice were maintained on a C57BL/6 background in an access-controlled animal facility at Sanford Research staffed by trained animal technicians, support staff and facility veterinarians. Health checks were performed by laboratory and facility staff at least once daily, including on weekends and holidays. Mice were maintained in isolated caging with pressurized ventilated rack systems to promote animal health. Breeding pairs were established at 2-3 months of age and retired by 9 months of age. All mice were maintained in a normal light-dark cycle of 12 h/12 h and fed on standard diet *ad libitum*. Both male and female mice were used for all experiments. Littermates were used for all experiments. All animal work was reviewed and approved by the Institutional Animal Care and Use Committee at Sanford Research (protocol #186-09-24B).

### Isolation and culture of astrocytes and microglia

Mouse astrocytes and microglia were purified from P2-P5 cerebral cortices and cultured as previously described (Parviainen et al., 2017). Cortices were dissociated with 0.25% trypsin-EDTA (Thermo Fisher Scientific, 25200056) and then mechanically dissociated to generate a single-cell suspension. Following a series of centrifugation steps, the cell pellet was resuspended in fresh medium prior to single-cell purification using a 70 µm cell strainer (Thermo Fisher Scientific, 22-363-548). Cells were plated onto poly-D-lysine (25 µg/ml, Sigma-Aldrich, P7886)-coated plates and cultured for 21 days to allow for maturation. Glial cells were cultured in Dulbecco's Modified Eagle Medium (DMEM)/Nutrient Mixture F-12 with L-glutamine (Life Technologies, 11320033) supplemented with 10% fetal bovine serum (Cytiva HyClone, SH3039603), 1% penicillin-streptomycin (10,000 U/ml, Life Technologies, 15140122). On day 21, the cells were placed on a cell shaker at 180 rpm overnight to detach the microglia. The supernatant containing microglia was removed to be cultured separately. Purified microglia were cultured in RPMI 1640 Medium with GlutaMAX (Thermo Fisher Scientific, 61870036) supplemented with 5% fetal bovine serum, 1% penicillin-streptomycin (10,000 U/ml), 10 ng/ml macrophage colony-stimulating factor (M-CSF, Peprotech, 315-02) and 10 ng/ml granulocyte macrophage colony-stimulating factor (GM-CSF, Peprotech, 315-03). Human primary cortical astrocytes (ScienCell Research Laboratories, 1800-5, lot #10869) were maintained in a commercially available medium (ScienCell Research Laboratories, 1801) as per manufacturer instructions. Cultures were confirmed to be free of mycoplasma contaminants every 3-4 months. To force cells to utilize endogenous cholesterol synthesis mechanisms and prevent lipoprotein-mediated internalization from the culture medium, the medium containing FBS was removed and glia were cultured in 10% lipoprotein-deficient serum (LPDS) for 7 days. LPDS was prepared by our laboratory as previously described (Anderson et al., 2021). LPDS preparations were lot tested to confirm cholesterol depletion and maintenance of cell viability and growth. All research was approved by the Sanford Research Institutional Biosafety Committee (#2019101).

### Pharmacological inhibition of cholesterol biosynthesis

Control astrocytes were treated with the following small molecules for 72 h to disrupt cholesterol synthesis at varying synthetic steps: AY9944 (5 µM; DHCR7 inhibitor; Cayman Chemical, 14611), U18666A (20 nM; DHCR24 inhibitor; Cayman Chemical, 10009085) and atorvastatin (2.5 µM; HMG-CoA reductase inhibitor; Cayman Chemical, 10493). All comparisons for statistical analyses were made relative to vehicle-treated controls (dimethyl sulphoxide, DMSO; Thermo Fisher Scientific, BP231-100) cultured in LPDS conditions.

### Immunocytochemistry

Cells were fixed in 4% paraformaldehyde (PFA; Electron Microscopy Sciences, 15714) for 20 min at room temperature and then permeabilized with 0.2% Triton X-100 (Sigma-Aldrich, 93443) for 20 min at room temperature. Blocking buffer consisting of 5% donkey or goat serum (Jackson ImmunoResearch Laboratories, 017-000-121 or 005-000-121) and 0.1% Triton X-100 was added to cells for 1 h prior to antibody incubation. The following primary antibodies were diluted in blocking buffer: chicken anti-GFAP (Novus Biologicals, NBP1-05198, lot 7529-11, 1:1000), mouse anti-S100β (Sigma-Aldrich/Millipore, S2532, lot 98917, 1:500), mouse anti-CD68 (Bio-Rad, MCA1957, lot 1708, 1:500), mouse anti-O4 (Novus Biological, MAB1326, lot HWW1117111, 1:500). Primary antibodies were visualized with Alexa Fluor-conjugated secondary antibodies (Life Technologies, A11001, A21437 and A31570; 1:500). CellMask Blue stain (Invitrogen, H32720, lot 2298095, 1:2500), Hoechst 33342 nuclear counterstain (Invitrogen, H3570, lot 1724829, 1:10,000) or BODIPY 505/515 (Invitrogen, D3921, lot 2301101, 1:2500) were incubated with the secondary antibodies where indicated.

### Immunohistochemistry

Animals were anaesthetized with CO_2_ and perfused with ice-cold PBS (Genesee Scientific, 25-508), then with ice-cold 4% PFA. Dissected brains were post-fixed in 4% PFA and then dehydrated in 20% sucrose (Sigma-Aldrich, 84097) overnight at 4°C. Brains were embedded in OCT (Thermo Fisher Scientific, 23-730-571) and 15 µm tissue sections were prepared with a cryostat (Leica, CM1850). Sections were rehydrated with PBS for 5 min at room temperature and then treated with a 2:1 ethanol/glacial acetic acid mixture for 5 min at −20°C. Sections were washed with PBS and permeabilized with 0.2% Triton X-100 for 20 min at room temperature. Blocking buffer consisting of 5% donkey or goat serum and 0.1% Triton X-100 was added to sections for 1 h prior to antibody incubation. The following primary antibodies were diluted in blocking buffer: chicken anti-GFAP (Novus Biological, NBP1-05198, lot 7529-11, 1:1000), rabbit anti-Iba1 (BioCare Medical, CP290A, lot 092221A, 1:500) and mouse anti-CD68 (Bio-Rad, MCA1957, lot 1708, 1:500). Primary antibodies were visualized with Alexa Fluor-conjugated secondary antibodies (Life Technologies, A21437, A31572 and A11001; 1:500). Hoechst 33342 (1:10,000) was incubated with the secondary antibodies.

### CX7 high-content analysis

Astrocytes were plated in 24-well glass-bottomed plates (CellVis, P24-0-N) coated with poly-D-lysine (25 µg/ml) at a density of 52,500 cells/cm^2^. Cells were fixed 48 h after plating and immunolabeled as described above. Astrocytes were imaged using the CellInsight CX7 High Content System (Thermo Fisher Scientific). Analyses of GFAP intensity and cell area were completed using HCS Compartmental Analysis software (Version 4, Thermo Fisher Scientific). Analyses of BODIPY and CD68 puncta were analyzed using HCS Spot Detection software (Version 4, Thermo Fisher Scientific).

### GC/MS sterol analysis

Sterol analysis of cell pellets was performed as previously described (Anderson et al., 2021). Astrocyte cell pellets were flash frozen, reconstituted in water and lysed by successive freeze/thaw cycles in a 37°C bead bath. Saponification buffer containing 7% KOH (Thermo Fisher Scientific, SP236) in 92% ethanol with 10 µg/ml coprostan-3-ol (Cayman Chemical, C7578) was added to the cell lysate. After saponification at 60°C, the aqueous phase was extracted with ethyl acetate by vortexing and centrifugation at 1100 ***g*** for 5 min. The organic phase was then extracted and concentrated to dryness by heating at 50°C under constant nitrogen flow. The residue was then dissolved in 50 µl pyridine (CHROMASOLV Plus, Sigma-Aldrich, 60-045-737) and sterols were derivatized in 50 µl N,O-bis(trimethylsilyl) trifluoroacetamide with 1% trimethylchlorosilane (BSTFA+1% TMCS, Thermo Fisher Scientific, TS-38831) for 1 h at 60°C. Samples were analyzed by automatic injection of the derivatized sterol mixture into an Agilent 7890 GC using a split injection port (4 mm ID×78.5 mm quartz wool liner, Restek, 23309) leading to a 0.18 mm ID×20 m 1,4-bis(dimethylsiloxy)phenylene dimethyl polysiloxane column (Restek, 43602). Helium was used as a carrier gas at a linear rate of 46.9 cm/sec. After 0.5 min at 170°C, the oven temperature was raised to 250°C at 18°C/min, then to 280°C at 3°C/min, and finally to 320°C at 20°C/min and held for 7 min. An Agilent 5977B mass spectrometer was operated in the electron impact mode at 70 eV with an ion source temperature of 275°C. Analysis was performed using MassHunter software. Identification of trimethylsilyl ethers of natural sterols was determined through comparison to commercially available standards for cholesterol, 7-DHC, lathosterol and desmosterol (Avanti Polar Lipids), as well as comparison to MS spectra through the National Institute of Standards and Technologies Standard Reference Database when available. Retention times and mass to charge (*m/z*) ratios are summarized in Table S1. For peak quantitation, sterol abundance was normalized to both the internal standard (coprostanol) and protein concentration (MicroBCA Protein Assay, Thermo Fisher Scientific, 23235). Data is presented relative to control samples. Trimethylsilyl derivatives of sterols exhibiting abundance <3% were excluded from analysis as previously described (Anderson et al., 2021).

### qRT-PCR

RNA was isolated from flash-frozen cell pellets using the Total RNA Kit (Omega BioTek, R6834-01) or the Quick-RNA Microprep Kit (Zymo Research, R1050), and cDNA was synthesized from 1 µg RNA using the High Capacity cDNA Reverse Transcription Kit (Thermo Fisher Scientific, 43-688-14) according to the manufacturer's protocol. qRT-PCR was performed using primers (Tables S2 and S3) and Forget-Me-Not EvaGreen qPCR Master Mix (Biotium, 31042) on an Applied Biosystems 7500 real-time PCR system following manufacturer-recommended protocols.

### Western blotting

Brains from P42 mice were lysed in 500 µl of lysis buffer with protease inhibitor (Thermo Fisher Scientific, A32955). Quantitation of protein was performed using Pierce BCA Protein Assay Kit (Thermo Fisher Scientific, PI23227). Approximately 20 µg of protein was loaded per lane of 4-20% Mini-PROTEAN precast protein gels (Bio-Rad, 4561094). Gels were blotted onto PVDF membranes (Thermo Fisher Scientific, IPVH00010), which were blocked in 5% milk and incubated with primary antibodies (anti-GLAST1, Novus Biologicals, NB100-1869, lot F-1, 1:1000; α-tubulin, Santa Cruz Biotechnology, sc-32293, lot C1120, 1:1000) diluted in 5% milk overnight at 4°C. The membranes were then incubated with the corresponding secondary antibodies (Jackson ImmunoResearch, 711-035-151 and 711-035-152, 1:10,000) for 1 h at room temperature and visualized on a LI-COR Odyssey FC Imager using Immobilon Classico Western HRP substrate (Millipore Sigma, WBLUC0100). Protein molecular mass was verified using the Precision Plus Protein Kaleidoscope prestained protein ladder (Bio-Rad, 1610375).

### Calcium imaging and analysis

Astrocytes were plated in 35 mm Fluorodish cell-culture dishes (World Precision Instruments, FD35-100) coated with poly-D-lysine (25 µg/ml) at a density of 11,500 cells/cm^2^. Live imaging was performed 48 h after plating (37°C, 5% CO_2_). Cells were rinsed with phenol-free DMEM/F12 (Life Technologies, 11039021) and incubated with 2.5 µM Fluo-4 AM (Thermo Fisher Scientific, F14217, lot 1890513) to label free intracellular calcium for 60 s at room temperature before adding fresh phenol-free DMEM/F12. Baseline activity was measured for 2 min. Cells were then stimulated with either 3 µM ATP (Thermo Fisher Scientific, 50-904-9890) or 3 µM L-glutamic acid (Sigma-Aldrich, 49621) for 2 min as previously described (Lundin et al., 2018). Videos were exported to stacked TIFF files and analysis was performed using ImageJ software (v2.1.0). Regions of interest (ROIs) were drawn for the soma of each cell fully within the field. The multi-measure function was used to measure the mean intensity (normalized to ROI area) for each frame. Calcium imaging analysis included three biological replicates per group with at least ten cells being analyzed for each replicate.

### *In situ* hybridization of target RNA and immunohistochemistry

Animals were anaesthetized with CO_2_ and perfused with ice-cold PBS, then with ice-cold 4% PFA. Dissected brains were post-fixed in 4% PFA and then dehydrated in 20% sucrose overnight at 4°C. Brains were embedded in OCT and 15 μm tissue sections were prepared with a cryostat (Leica, CM1850). Slides were washed once in PBS to remove residual OCT and baked at 60°C for 30 min in a hybridization oven. Slides were post-fixed in 4% PFA at 4°C for 15 min before being dehydrated using a series of ethanol washes (50%, 70%, 100% and 100%) and dried at room temperature for 5 min. Sections were then incubated in hydrogen peroxide (Advanced Cell Diagnostics, 322330) for 10 min at room temperature and washed twice with water. Following a 5 min antigen retrieval, slides were transferred to 100% ethanol for 3 min and dried for 5 min at room temperature. Slides were then treated with RNAscope Protease III (Advanced Cell Diagnostics, 322340) for 30 min in a 40°C hybridization oven. Sections were washed in water before proceeding with the RNAscope fluorescent assay v2 (Advanced Cell Diagnostics, 323110) as per the manufacturer's instructions. RNAscope probes (Advanced Cell Diagnostics, 511541 and 572491-C2) were applied to the sections for 2 h in a hybridization oven. Following the recommended amplification and wash steps, the *Ptx3* signal in the C1 channel was developed with 1:1200 dilution of the TSA Plus Fluorescein Kit (Perkin Elmer, NEL741E001KT) and the *Gbp2* signal in the C2 channel was developed with 1:750 dilution of the TSA Plus Cy5 Kit (Perkin Elmer, NEL745001KT). The *in situ* hybridization assay was combined with immunofluorescence assay following the manufacturer's protocol. Slides were blocked in TBS with 0.01% Tween 20 (TBS-T) and 0.1% BSA for 30 min at room temperature, followed by incubation with chicken anti-GFAP (Novus Biologicals, NBP1-05198, 1:500) overnight at 4°C. Slides were then washed with TBS-T and incubated with Alexa Fluor 555 goat anti-chicken secondary antibody (Life Technologies, A-21437, 1:500) and Hoechst 33342 (1:10,000) for 1 h at room temperature.

### *In situ* hybridization of target RNA and immunocytochemistry

Astrocytes were washed with 1× PBS and fixed with 10% neutral buffered formalin (Thermo Fisher Scientific, 51601) for 30 min at room temperature. Following fixation, cells were washed in 1× PBS and dehydrated using a series of ethanol washes (50%, 70%, 100% and 100%) for 5 min each at room temperature. Cells were then rehydrated with ethanol (70% and 50%) for 2 min at room temperature before being washed with 1× PBS. Following rehydration, cells were treated with hydrogen peroxide for 10 min at room temperature to block endogenous peroxidase activity, and then washed twice with distilled water. Cells were permeabilized with 1:15 dilution of Protease III (Advanced Cell Diagnostics, 322340) in PBS for 10 min in a hybridization oven (40°C, humidified environment). Cells were then washed twice in 1× PBS before proceeding with RNAscope fluorescent assay v2 as per the manufacturer's instructions. Briefly, RNAscope probes (Advanced Cell Diagnostics, 511541 and 572491-C2) were applied to the sections for 2 h in a hybridization oven. Following the recommended amplification and wash steps, the *Ptx3* signal in the C1 channel was developed with 1:1200 dilution of the TSA Plus Fluorescein Kit (Perkin Elmer, NEL741E001KT) and the *Gbp2* signal in the C2 channel was developed with 1:750 dilution of the TSA Plus Cy5 Kit (Perkin Elmer, NEL745001KT). The *in situ* hybridization assay was combined with immunofluorescence assay following the manufacturer's protocol. For this, slides were blocked in TBS-T with 0.1% BSA for 30 min at room temperature, followed by incubation with chicken anti-GFAP (Novus Biologicals, NBP1-05198, 1:500) overnight at 4°C. Cells were then washed with TBS-T, and incubated with Alexa Fluor 555 goat anti-chicken secondary antibody (Life Technologies, A-21437, 1:500) and Hoechst 33342 (1:10,000) for 1 h at room temperature.

### Statistical analysis

All statistical analyses were performed using GraphPad Prism 8.0.2 (GraphPad Software, CA, USA). Assuming equal variance, data were analyzed by unpaired two-tailed *t*-test or one-way ANOVA and post hoc Dunnett's *t*-test for multiple comparisons relative to LPDS control groups. *P*<0.05 was accepted as significant. *P<0.05; **P<0.01; ***P<0.001; ****P<0.0001. Statistical details for each experiment can be found within the corresponding figure legends.

## Supplementary Material

10.1242/dmm.049843_sup1 Supplementary information
