# Supplementary material for: Sterol dysregulation in Smith–Lemli–Opitz syndrome causes astrocyte immune reactivity through microglia crosstalk
Source: Dis Model Mech. 2022 Dec 16;15(12):dmm049843. doi: 10.1242/dmm.049843 (PMC10655813; doi:10.1242/dmm.049843)
Supplement: Supplementary information [file dmm-15-049843-s1.pdf]

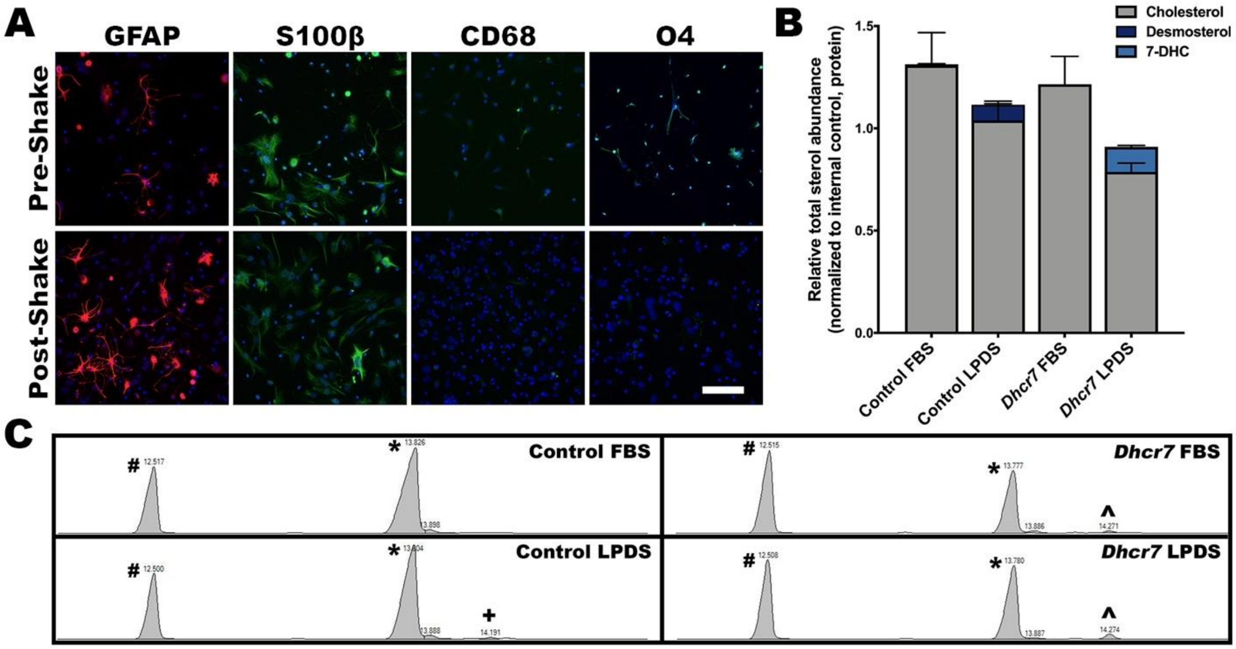

Fig. S1. Validation of primary astrocyte purity and biochemical defects. (A) Immunolabeling of primary cultures before and after purification of astrocytes. GFAP (red) and S100β label astrocytes. CD68 (green) labels microglia. O4 (green) labels oligodendrocytes. Hoechst nuclear counterstain (blue) also shown. Scale bar: 100 μm. (B) GC/MS analyses of primary control and *Dhcr7* astrocytes in cholesterol replete or cholesterol depleted conditions. (C) Representative GC/MS chromatograms of control and *Dhcr7* astrocytes cultured in FBS or LPDS. Data show the mean ± s.e.m. N = 3 biological replicates. (# = coprostanol, ∗ = cholesterol, + = desmosterol, ∧ = 7-dehydrocholesterol).

Disease Models & Mechanisms • Supplementary information

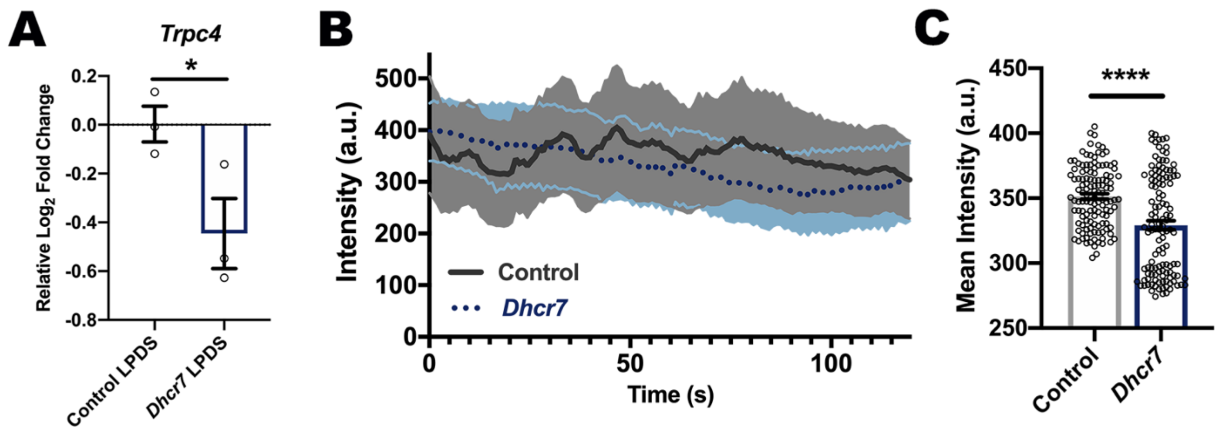

Fig. S2. *Dhcr7* astrocytes display reduced baseline calcium activity and response to ATP stimulation. (A) *Trpc4* transcript expression is reduced in *Dhcr7* astrocytes upon LPDS culture. Data show the mean ± s.e.m. (*P<0.05; two-tailed unpaired t-test. N = 3 biological replicates; each data point represents the average of three technical replicates). (B) *Dhcr7* astrocytes exhibit reduced calcium response to ATP stimulation compared to control astrocytes. (C) Mean calcium intensity following ATP stimulation was reduced in *Dhcr7* astrocytes. Data show the mean ± s.e.m. (*P<0.05; ****P<0.0001; two tailed unpaired *t*-test. N = 360 from three recordings per group; each data point represents the average intensity of the replicates over time).

Disease Models & Mechanisms • Supplementary information

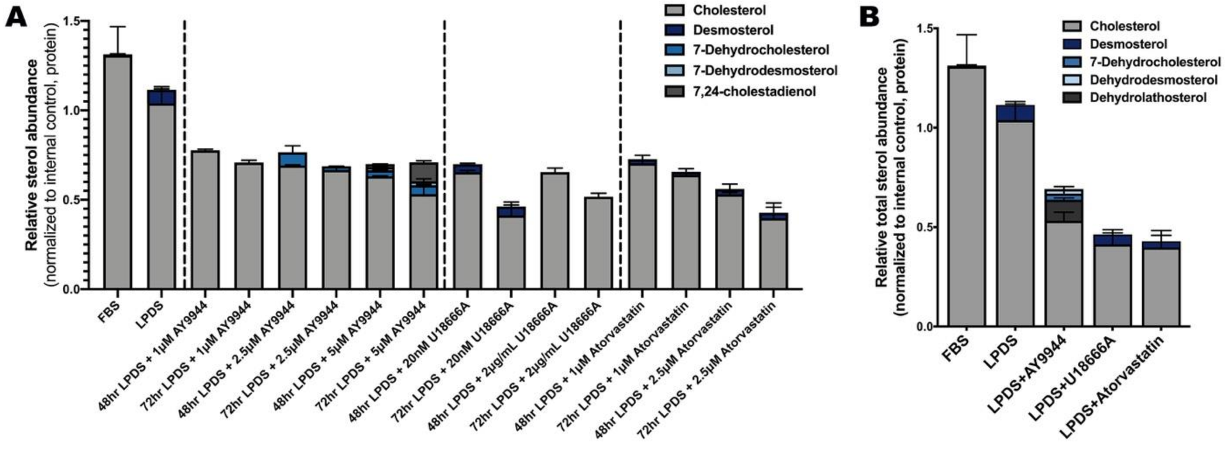

Fig. S3. Pharmacological inhibition of cholesterol synthesis and sterol profile changes. (A) Quantified GC/MS analyses of sterol content following dose response assay in control astrocytes treated with inhibitors of cholesterol biosynthesis. (B) Quantified GC/MS analyses of sterol content in control astrocytes treated with inhibitors of cholesterol biosynthesis demonstrates cholesterol depletion and accumulation of alternate sterols. Data show the mean ± s.e.m. N = 3 biological replicates per group.

Disease Models & Mechanisms • Supplementary information

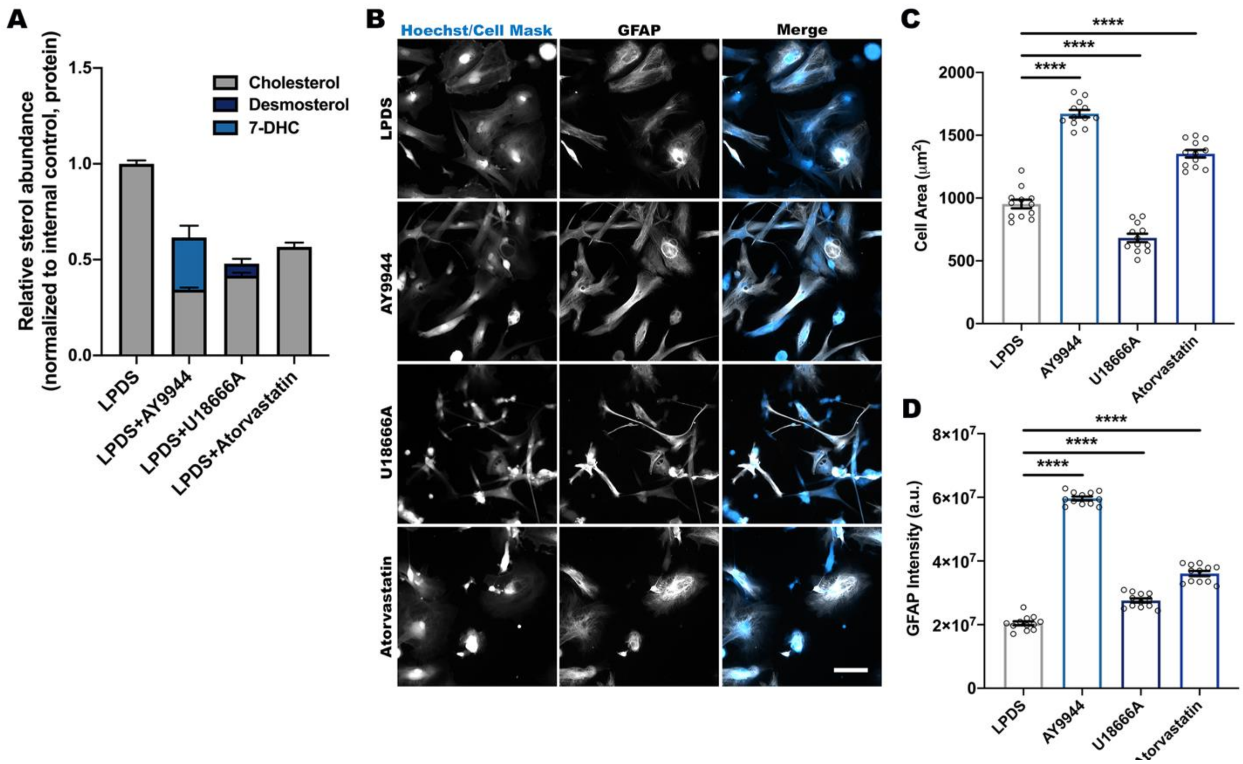

Fig. S4. Pharmacological inhibition of cholesterol synthesis in human astrocytes induces morphological change consistent with reactivity. (A) Quantified GC/MS analyses of sterol content in human astrocytes treated with inhibitors of cholesterol biosynthesis shows substantial reduction of cholesterol levels accompanied by accumulation of sterol precursors (N = 3 biological replicates). (B) Hoechst nuclear counterstain and CellMask (blue) with GFAP (white) immunolabeling of human primary astrocytes. (C,D) Treatment of control human astrocytes with cholesterol biosynthesis inhibitors induces cellular hypertrophy and increased GFAP intensity. Scale bar: 100 μm. Data show the mean ± s.e.m. (****$P< 0.0001$; one-way ANOVA and Dunnett's test versus LPDS control. N = 12 wells from three biological replicates, mean of 40 images per well).

Disease Models & Mechanisms • Supplementary information

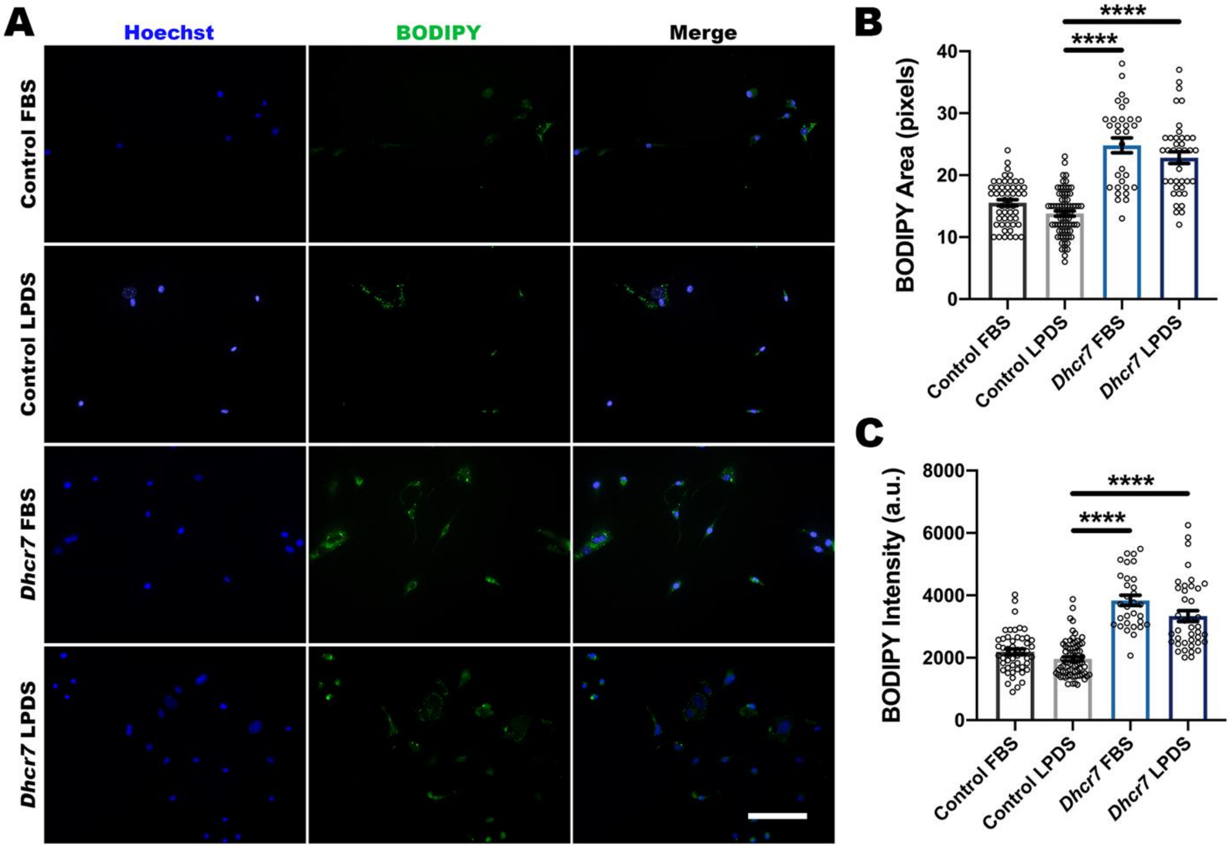

Fig. S5. *Dhcr7* microglia exhibit enhanced lipid droplet accumulation. (A) Hoechst nuclear counterstain (blue) and BODIPY (green) immunolabeling of control and *Dhcr7* primary microglia. (B,C) Analyses of control and *Dhcr7* microglia shows increased BODIPY spot area and intensity in *Dhcr7* microglia. Scale bar: 100 μm. Data show the mean ± s.e.m. (****$P < 0.0001$; one-way ANOVA and Dunnett's test versus LPDS control. Average of 48 cells per group from three biological replicates, five images per replicate; each data point represents average spot area or intensity per cell).

Disease Models & Mechanisms • Supplementary information

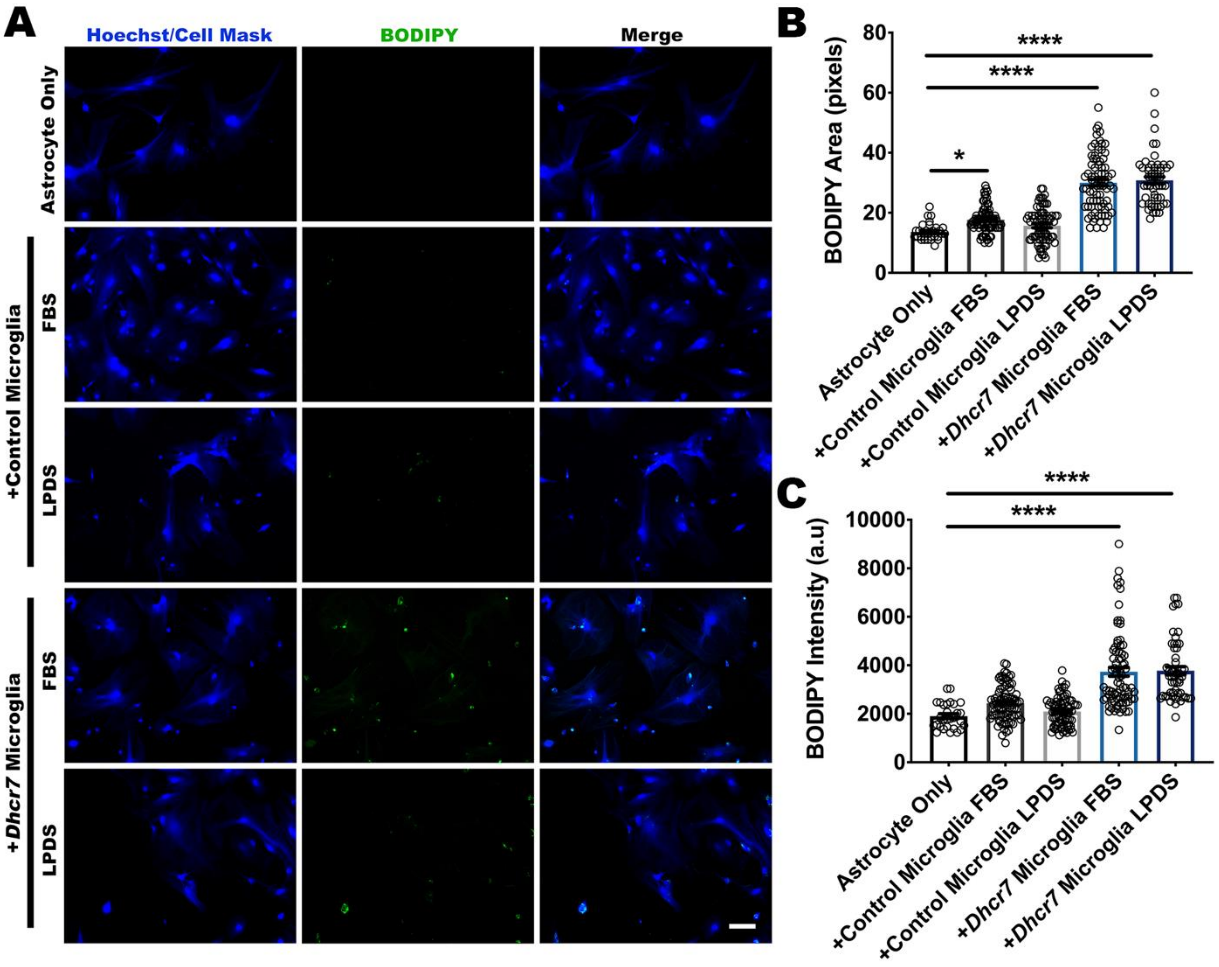

Fig. S6. *Dhcr7* microglia induce lipid droplet accumulation in human primary astrocytes. (A) Hoechst nuclear counterstain and CellMask (blue) with BODIPY (green) immunolabeling of control and *Dhcr7* microglia. (B,C) Control human astrocytes co-cultured with control or *Dhcr7* mouse microglia exhibit increased BODIPY spot area and intensity when cultured with *Dhcr7* microglia. Scale bar: 100 μm. Data show the mean ± s.e.m. (*P< 0.05; ****P< 0.0001; one-way ANOVA and Dunnett's test versus astrocyte only control; Average of 64 cells per group from three biological replicates, five images per replicate; each data point represents average spot area or intensity per cell).

Disease Models & Mechanisms • Supplementary information

Table S1. Chromatographic and mass spectral parameters of sterols detected by GC/MS. Following isolation from astrocyte cultures, sterols were derivatized with trimethylsilyl (TMS) to ethers. After separation on a Rxi-5 Sil column (Restek, 43602), the above retention times and major fragment ions (m/z) were detected.

Sterol, sterol derivatives	Retention time (min)	Molecular ion m/z (Da)	Base peak m/z (Da)	Spectral ions m/z (Da)
Coprostanol TMS	12.5-12.6	Not detected	370	403, 355, 215
Cholesterol TMS	13.7-13.8	458	329	443, 368, 353, 129
7,24-cholestadienol TMS	13.9-14.0	456	351	456, 238, 182
Desmosterol TMS	14.2-14.3	456	129	441, 366, 343, 327
7-dehydrocholesterol TMS	14.3-14.6	456	351	441, 366, 325
7-dehydrodesmosterol TMS	14.6-14.7	454	454	439, 369, 349

Table S2. Primer sequences utilized to detect and quantify mouse transcript expression by qRT-PCR.

ID	Forward Sequence	Reverse Sequence	Product Size
Vim	AGACCAGAGATGGACAGGTGA	TTGCGCTCCTGAAAAACTGC	169
Gfap	AGAAAGGTTGAATCGCTGGA	CGGCGATAGTCGTTAGCTTC	299
Cxcl10	CCCACGTGTTGAGATCATTG	CACTGGGTAAAGGGGAGTGA	211
Fbln5	CTTCAGATGCAAGCAACAA	AGGCAGTGTCAGAGGCCTTA	281
Fkbp5	TATGCTTATGGCTCGGCTGG	CAGCCTTCCAGGTGGACTTT	194
Ptx3	AACAAGCTCTGTTGCCCATT	TCCCAAATGGAACATTGGAT	147
Gbp2	GGGGTCACTGTCTGACCACT	GGGAAACCTGGGATGAGATT	285
S100a10	CCTCTGGCTGTGGACAAAAT	CTGCTCACAAGAAGCAGTGG	238
Cd14	GGACTGATCTCAGCCCTCTG	GCTTCAGCCCAGTGAAAGAC	232
Il-1α	CGCTTGAGTCGGCAAAGAAAT	CTTCCCGTTGCTTGACGTTG	271
Il-1β	TGCCACCTTTTGACAGTGATG	TGATGTGCTGCTGCGAGATT	138
Tnfα	TGTGCTCAGAGCTTTCAACAA	CTTGATGGTGGTGCATGAGA	88
C1q	TCTGCACTGTACCCGGCTA	CCCTGGTAAATGTGACCCTTTT	232
Arg1	TTTTAGGGTTACGGCCGGTG	CCTCGAGGCTGTCCTTTTGA	146
Ccl2	CACTCACCTGCTGCTACTCA	GCTTGGTGACAAAAACTACAGC	117
Marco	TTCTGTCGCATGCTCGGTTA	CAGATGTTCCCAGAGCCACC	71
Msr1	CCAGCAATGACAAAAGAGATGACA	CTGAAGGGAGGGGCCATTTT	150
Ppia	GTCTCCTTCGAGCTGTTTGC	GCGTGTAAAGTCACCACCCT	150

Disease Models & Mechanisms • Supplementary information

Table S3. Primer sequences utilized to detect and quantify human transcript expression by qRT-PCR.

ID	Forward Sequence	Reverse Sequence	Product Size
VIM	GGACCAGCTAACCAACGACA	TCCTCCTGCAATTTCTCCCG	93
GFAP	AGATCCACGAGGAGGAGGTT	TCATACTGCGTGCGGATCTC	124
CXCL10	CCACGTGTTGAGATCATTGCTAC	ATCGATTTTGCTCCCCTCTGG	149
FBLN5	TTCTTCTCGCCTTCGCATCT	ATTCGTGCACTGTGCCTGT	127
FKBP5	GCGGCGACAGGTTCTCTA	CTCCCTGCTCAGCAACAGT	96
PTX3	GCCGCGGTGCTAGAGG	TAAAATAGCTGTTTCACAACCTGCC	105
GBP2	GGATCTCTGATCTGGGGAACAAC	CCACCACAGGCTGCGTAAT	150
S100A10	TCGCTGGGGATAAAGGCTAC	CCAGAGGGTCTTTTTGATTTTCCA	96
CD14	GGAAGACTTATCGACCATGGAGC	AGACGCAGCGGAAATCTTCA	122
GAPDH	AATTCCATGGCACCGTCAAG	ATCGCCCCACTTGATTTTGG	104

Disease Models & Mechanisms • Supplementary information
